# CIDER: an interpretable meta-clustering framework for single-cell RNA-seq data integration and evaluation

**DOI:** 10.1186/s13059-021-02561-2

**Published:** 2021-12-13

**Authors:** Zhiyuan Hu, Ahmed A. Ahmed, Christopher Yau

**Affiliations:** 1 Ovarian Cancer Cell Laboratory, MRC Weatherall Institute of Molecular Medicine, University of Oxford, Oxford, OX3 9DS UK; 2 Nuffield Department of Women’s and Reproductive Health, University of Oxford, Oxford, OX3 9DU UK; 3 Current Address: MRC Weatherall Institute of Molecular Medicine, Radcliffe Department of Medicine, University of Oxford, Oxford, OX3 9DS UK; 4 Division of Informatics, Imaging and Data Sciences, Faculty of Biology Medicine and Health, The University of Manchester, Manchester, M13 9PT UK; 5 Alan Turing Institute, London, NW1 2DB UK; 6 Health Data Research UK, Gibbs Building, 215 Euston Road, London, NW1 2BE UK

**Keywords:** Clustering, Single-cell RNA-Seq, Confounding factors

## Abstract

Clustering of joint single-cell RNA-Seq (scRNA-Seq) data is often challenged by confounding factors, such as batch effects and biologically relevant variability. Existing batch effect removal methods typically require strong assumptions on the composition of cell populations being near identical across samples. Here, we present CIDER, a meta-clustering workflow based on inter-group similarity measures. We demonstrate that CIDER outperforms other scRNA-Seq clustering methods and integration approaches in both simulated and real datasets. Moreover, we show that CIDER can be used to assess the biological correctness of integration in real datasets, while it does not require the existence of prior cellular annotations.

**Supplementary Information:**

The online version contains supplementary material available at 10.1186/s13059-021-02561-2.

## Background

The widespread adoption of single-cell RNA sequencing (scRNA-Seq) as a modality for the investigation of functional cellular heterogeneity means it is now routine for multiple datasets to be generated from the same type of tissues and organs across a number of individuals. Integration of multiple scRNA-Seq datasets can provide more comprehensive interpretations by borrowing information across experiments and even species [1]. However, the data from multiple experiments are often confounded by inter-batch or inter-donor variability.

Existing clustering workflows can effectively identify cell populations in batch-effect-free datasets [2], by partitioning cells based on the inter-cell distance matrix computed from the expression data of high variance genes (HVGs) or the derived principal components. For example, SC3 constructs the distance matrix by applying Euclidean, Pearson, and Spearman metrics on the expression data of HVGs and transfers this distance matrix by principal component analysis (PCA) or graph Laplacian transformation, before consensus clustering [3]. RaceID computes the distance matrix in the same way as SC3 but provides more options of distance measures, including Kendall and proportionality [4]. Seurat v3 calculates Euclidean distances from the principal components and then infers the graph of shared nearest neighbors for the subsequent graph-based clustering, such as Louvain clustering [5]. However, distance measurements used by these workflows cannot effectively distinguish biological variation from the technical one and, thus, their performance is compromised in datasets confounded by batch effects or other variability caused by unwanted or unexplained factors.

In data confounded by batch effects, workflows combining batch correction or integration methods and downstream clustering algorithms are used to identify cell populations. Some existing batch correction and integration methods can efficiently correct the gene expression or dimensionality reduction spaces for visualization and other downstream analyses. For example, mutual nearest neighbors [6] (MNN) uses the cell pairs that are mutually nearest neighbors to compute a vector that aligns multiple batches into a common space, which is also incorporated in the Monocle3 pipeline [7]. Scanorama [8] also used the concept of MNNs to merge datasets with substantial improvement in the MNN search strategies. Seurat exploits canonical correlation analysis [9] (CCA) and reciprocal PCA [10] (RPCA) to compute a subspace and then used the identified MNNs, i.e., “anchors,” to correct the data. Harmony [11] iteratively diminishes batch effects in the PCA space by soft clustering across batches and then adjusting cell positions based on the global and dataset-specific cluster centroids. LIGER [12] exploits integrative non-negative matrix factorization to compute the factor loading matrix for cell type assignment. Combat [13] leverages the empirical Bayesian framework to derive the corrected gene expression matrix. Clustering on network of samples [14] (Conos) computes the cell-cell connection and downweights the intra-sample connections to construct a joint graph for downstream analysis. However, for the majority of integration methods, performance can vary substantially across data types and scenarios [15]. An additional limitation of the commonly used integration algorithms, e.g., CCA and Harmony, is that they work on the low-dimensional representation, which can be affected by the bias in the initial selection of HVGs and principal components. Furthermore, it is often difficult to determine why existing methods drive cells from different batches into the same cluster. This lack of explainability or interpretability can make it difficult to ascertain if integration has been successful.

To address this limitation, we recently introduced the use of meta-clustering to partition scRNA-Seq data from ovarian cancer fallopian tube epithelial cells confounded by structured batch effects and inter-patient variability [16]. This method was based on a functional hypothesis that cells from the same biological population (either cell type, subtype, or state) share a similar differential expression pattern, i.e., the differentially expressed genes (DEGs) having more weights to determine cell classes compared to other genes. Moreover, these DEGs are less affected by batch effects by regressing out the unwanted factors. In this work, we present a scalable version of this methodology and demonstrate its generalizable utility for wider application.

Here, we introduce a novel similarity metric based on Inter-group Differential ExpRession (IDER) and propose a workflow of Clustering by IDER (CIDER). We demonstrate that the performance of CIDER is comparable or superior to existing clustering workflows applied on uncorrected and batch-corrected datasets in a variety of scenarios for both simulated and real scRNA-Seq data. Furthermore, as IDER is a substantively different form of distance metric compared to those used in popular integration algorithms, we show that CIDER can also be used as a ground-truth-free evaluation metric for accurately identifying falsely integrated populations.

## Results

### Design of CIDER and proof-of-concept experiment

**Fig. 1:**
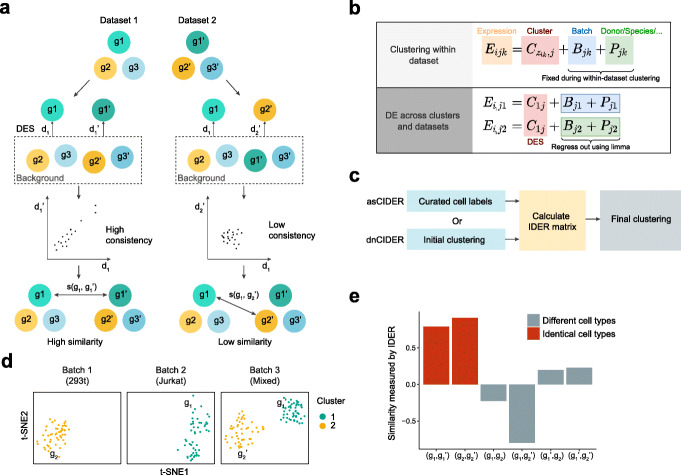
IDER metric accurately measures the biological similarity between cell groups. **A** Schematic diagram shows how the IDER metric measures the inter-group similarity. **B** The diagram shows the theoretical justification for CIDER. *E*_*ijk*_ denotes the expression level of gene *j* in cell *i* of batch *k*, *z*_*ik*_ cell assignment of cells of cell *i*, *C* cluster effect, *B* batch effect, and *P* patient effect or other biases. In the lower panel, *z*_*ik*_ is set to 1. **C** Schematic diagram of asCIDER and dnCIDER. **d** *t*-SNE plots show the cells from three batches of Dataset 1. Each subpanel represents a batch. Cells are colored by the population. Each batch-specific cluster is denoted by a label. **E** The IDER metric generated higher similarity between group pairs, (g_1_, g_1_’) and (g_2_, g_2_’), from identical cell types and lower similarity between group pairs from different cell types

The core of CIDER is the IDER metric, which can be used to compute the similarity between two groups of cells across datasets (Fig. 1A). IDER first identifies the differentially expressed signature (DES) for each group of cells against all other cells with the unwanted variables regressed out. Next, a similarity measure is computed by using the consistency of DESs between two groups across datasets. Differential expression in IDER is computed using the same principle as limma-trend [17], which was chosen from a collection of approaches for differential expression analysis based on a number of performance criteria (Additional file 1: Fig. S1A, B) [18].

CIDER is established on the hypothesis that the expression level contains the linear combination of the effects of cluster, batch, donor, platform, etc. (Fig. 1B). The within-dataset clustering enables the identification of the cluster effect (i.e., cell assignment) for a given dataset, as the confounding effect (e.g., batch effects, inter-donor variability, or inter-species variability) is a constant within the same dataset. Once the cell assignments are completed for all datasets, we use limma to regress out the confounding effects across datasets and identify consistent cluster effects, represented by DESs, from multiple datasets. Groups with a consistent cluster effect will be merged into one final cluster. In the workflow of CIDER, IDER is used to measure the pairwise inter-group similarity among the batch-specific initial clusters (Fig. 1C). These initial clusters can be either curated annotations or outputs from a clustering algorithm. The output of the IDER step, i.e., a similarity matrix, is used to merge the connected initial clusters into final cross-batch clusters. Depending on how the initial clusters were derived, we named the CIDER workflows as de novo CIDER (dnCIDER), where initial clusters were the output of a clustering algorithm, and assisted CIDER (asCIDER), where initial clusters were curated annotations of cell populations. These two scenarios were considered in our benchmarking because they are common in real-world usage.

We set about to test if the IDER metric could accurately estimate the cluster effects and regress out biases in data confounded by batch effects. As a proof-of-concept experiment, we applied it to a multiple cell line dataset (Dataset 1) [19], in which three batches corresponded to pure 293T cells, pure Jurkat cells, and a 50/50 mixture of both cell lines. The IDER metric was used to calculate the pairwise similarity among four groups from these three batches (Fig. 1D, Additional file 1: Fig. S2). We showed that the similarity computed by IDER was higher for the group pairs from the identical cell type compared to the pairs from different cell types (Fig. 1E), demonstrating the utility of IDER as a metric to identify cluster similarity across datasets when confounded by batch effects.

### Benchmarking clustering performance on simulated data

To test the accuracy of identifying populations, we benchmarked CIDER against other 12 workflows: nine workflows that combined integration approaches and clustering (Seurat-CCA [9], fastMNN [6], Scanorama [8], Harmony [11], LIGER [12], Combat [13], Monocle3 [7], Conos [14], and RPCA [10]) and three single-cell clustering approaches (Seurat v3-Louvain [5], SC3 [3], and RaceID [4]).

**Fig. 2:**
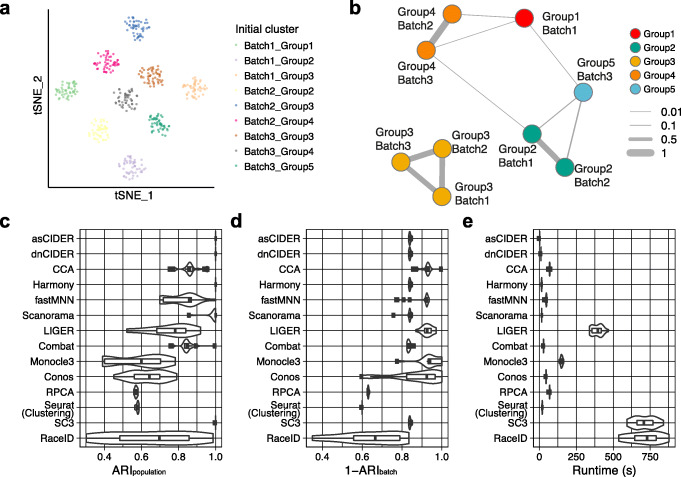
CIDER accurately identifies cross-batch populations. **A** *t*-SNE plot shows the nine initial clusters from three batches of the simulated dataset (Dataset 2). Cells are colored by initial clusters. **B** The graph network shows the similarity among initial clusters. Vertexes represent initial clusters, colored by populations. The width of edges represents the similarity levels. **C** Distribution of ARI_population_ for 20 replicates of simulated data across integration workflows and clustering algorithms. The *x*-axis denotes the workflow performance by calculating ARIs between cell populations and clustering results, and the *y*-axis denotes clustering and integration workflows. The whiskers from left to right in the boxplot represent the first quartile, the median, and the third quartile. **D**, **E** Distribution of 1-ARI_batch_ (**D**) and runtime (**E**) for 20 replicates of simulated data across integration workflows and clustering algorithms

We used a simulated dataset (Dataset 2, Additional file 1: Table S1) as a tailor-made, toy example, where three batches comprised non-identical compositions of populations (Additional file 1: Fig. S3A, B). The challenge is to be able to match clusters across batches, e.g., to identify that Group 3 cells (Yellow) exist across all three batches. In this scenario, the cross-batch similarity computed by CIDER correctly recognized the connection among initial clusters (Fig. 2A, B). In contrast, MNN and CCA overcorrected the batch effects, leading to the incorrect merging of disparate populations as previously reported [8] (Additional file 1: Fig. S3C-F). To quantitatively compare their performance, we computed the adjusted Rand indexes (ARIs) between cell labels and clustering results (ARI_population_) or the ARIs between batches and clustering results (ARI_batch_). Ideal performance is characterized by high ARI_population_ and low ARI_batch_ (i.e., high 1-ARI_batch_) such that cluster allocation is dominated by cell type and not batch, while in this scenario of unbalanced cell composition 1-ARI_batch_ close to 1 corresponds to overcorrection. The experimental replicates (*n* = 20) confirmed that CIDER robustly outperformed fastMNN and CCA in this scenario of non-identical cellular compositions (Fig. 2C). While Harmony, Scanorama, and SC3 could also identify the exact cell classes, like fastMNN and CCA, LIGER, Monocle3, and Conos also overcorrected the batch effects (Fig. 2D). For this dataset (*n* = 6000 cells), the running time (1.5 s and 10.9 s average) of asCIDER and dnCIDER was comparable to that of Harmony (15.5 s), Scanorama (15.9 s), and Seurat clustering (19.1 s) (Fig. 2E). While this was a toy example, this simple simulation illustrates the challenge of confounding effects. We next benchmarked CIDER on four real datasets.

### Benchmarking clustering performance on real data

**Fig. 3:**
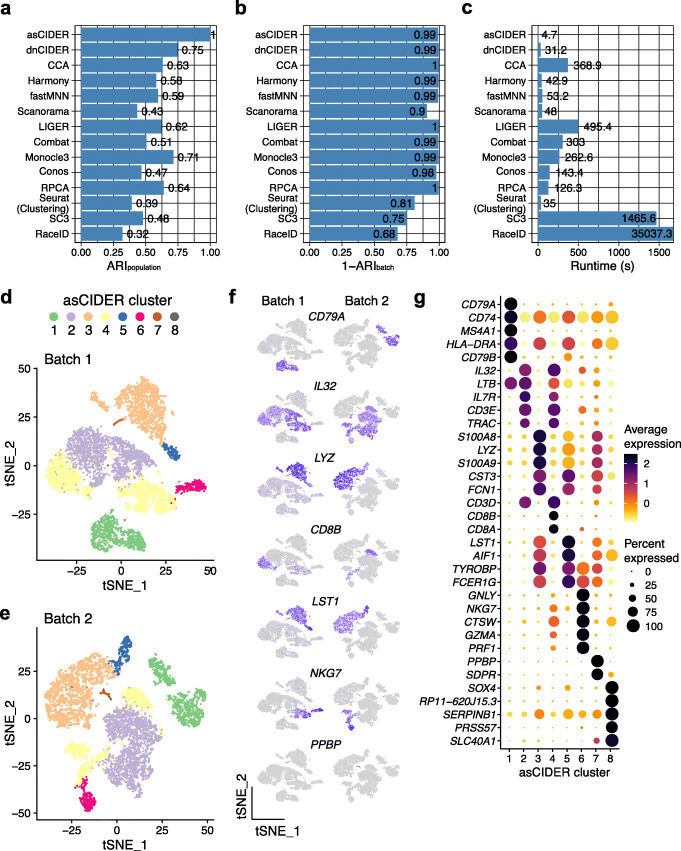
CIDER leads to precise cell classification in the joint PBMC data sequenced by two techniques (Dataset 3). **A**–**C** Distribution of ARI_population_ (**A**), 1-ARI_batch_ (**B**), and runtime (**C**) across integration workflows and clustering algorithms. **D** *t*-SNE plot of cells from Batch 1, colored by asCIDER clustering results (final clusters). **E** *t*-SNE plot of cells from Batch 2. **F** *t*-SNE plots of cells show expression levels of marker genes in two batches. **G** Average expression levels and percentages of expression of top marker genes across asCIDER clusters. The size of dots denotes the percentage of expression, and the color represents the average expression level

We next tested CIDER with Dataset 3 of human peripheral blood mononuclear cells (PBMCs) [19]. Cells were annotated into nine cell types and subtypes, namely B cell, CD4 T cell, CD8 T cell, hematopoietic stem cell (HSC), megakaryocyte, CD14 monocyte, FCGR3A monocyte, natural killer (NK) cell, and plasmacytoid dendritic cell [20]. Cells of this dataset were sequenced by either of two techniques (10x 3’ and 5’ single-cell gene expression), which we termed Batch 1 and Batch 2, respectively. The uncorrected space suggested that the data were confounded by batch effects (the variability introduced by techniques in this scenario), which forced a cognate cell population into more than one cluster (Additional file 1: Fig. S4A, B). We set the technique effect as the unwanted variable and regressed it out from the derived DES, which eliminated the influence of technique variability on the inter-group similarity matrix and the results of subsequently final clustering. Both dnCIDER and asCIDER outperformed other batch correction and clustering workflows regarding the accuracy of identifying populations (Fig. 3A). The meta-clustering workflows also overcame the effect of techniques, while the accuracy of sole clustering methods (Seurat clustering, SC3, and RaceID) was interfered as implied by the lower values of 1-ARI_batch_ (Fig. 3B). CIDER also had the shortest runtime in this dataset of moderate size (*n* = 14,876 cells) compared to other benchmarked methods (Fig. 3C). Because the dnCIDER clustering results have not been annotated according to biological functions, the results of asCIDER are used as an example to elucidate its biological relevance and interpretability for this dataset and the following ones. Beyond achieving joint clustering, asCIDER could reveal the underlying relationships among initial clusters via a network graph (Additional file 1: Fig. S4C, D). The cliques in the network graph suggested a hierarchical structure of cell populations. It not only presented the binary relationship, i.e., which initial clusters should be merged, but also quantified the strength of agreement, i.e., IDER-based similarity, among homogenous and heterogeneous populations. In addition to showing the connections, it revealed the relationships between heterogeneous populations. For example, CD4 and CD8 T cell populations, CD14 and FCGR3A monocyte populations, shared high pairwise similarity. The clustering results of CIDER methods, e.g., asCIDER, could be visualized in the unaligned low-dimensional space (Fig. 3D, E). In the downstream analysis, we regressed out the technical variability and identified the cluster-specific marker genes (Fig. 3F, G).

**Fig. 4:**
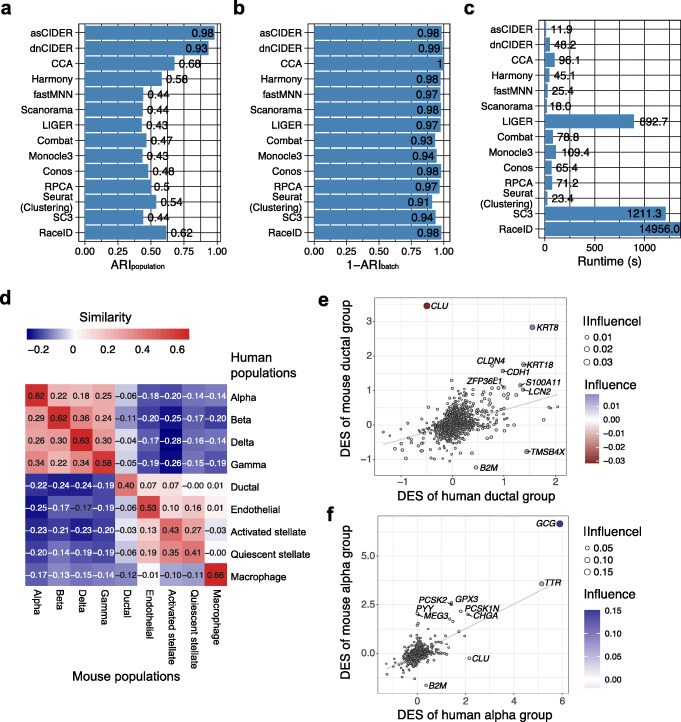
CIDER accurately identifies clusters between human and mouse samples in the cross-species pancreas data (Dataset 4). **A**–**C** Distribution of ARI_population_ (**A**), 1-ARI_batch_ (**B**), and runtime (**C**) across integration workflows and clustering algorithms. **D** Heatmap shows the inter-group similarity between mouse populations (*x*-axis) and human ones (*y*-axis). Cells are colored by the similarity levels, as shown by the numbers. **E** Scatter plot shows genes driving the similarity and dissimilarity between the human ductal group and the mouse ductal group. The *x*- and *y*-axes denote the DESs in humans and mice. Each dot is a gene, colored and sized by the influence and its abstract value. The gray line is the linear regression line for reference. Genes with the ten largest abstract values of influence are labeled. **F** Scatter plot shows genes driving the similarity and dissimilarity between the human alpha group and the mouse alpha group

Given interest in cross-species comparative analysis, we benchmarked CIDER on Dataset 4 that contains both human and mouse pancreatic data (Additional file 1: Fig. S5A, B) [21]. This dataset is composed of 2 mouse samples and 4 human samples, resulting in the structured combination of species effect and donor effect. CIDER was aimed to regress out the species effect, in which case the donor effect was treated as the nesting variable in the regression model. CIDER workflows outperformed other pipelines regarding the accuracy of identifying cell classes (ARI_population_) (Fig. 4A). With respect to the capability to correct batch effects (1-ARI_batch_), CIDER workflows were comparable to the other integration methods ranging between 0.97 and 1, except Combat and Monocle3, which had lower 1-ARI_batch_ (0.93 and 0.94, respectively) (Fig. 4B). Moreover, asCIDER cost the least amount of processing time, while the runtime of dnCIDER was slightly longer than Scanorama, fastMNN, and Harmony (Fig. 4C). Dataset 4 (*n* = 10,127) has fewer cells than Dataset 3 (*n* = 14,876). CIDER took longer to process Dataset 4 than Dataset 3 because its running time is approximately associated with the numbers of batch-specific clusters. In addition to identifying cell assignment, the asCIDER result revealed that the between-species similarity was inconsistent across cell types (Fig. 4D). Unlike methods based on low-dimensional space, the gene-level analysis of CIDER empowered its explainability by delineating how various genes contributed to inter-group similarity. The influence of individual genes was derived by the Fisher *z*-transformation. Positive values of influence indicated the affirmative contribution to similarity, while negative values denoted the contribution to dissimilarity. For example, the inter-species similarity (0.40) of the ductal cell population was suppressed by the existence of negative-influence genes, e.g., *CLU*, *TMSB4X*, and *B2M* (Fig. 4E). Yet the top positive-influence genes, e.g., *KRT8* and *KRT18*, were the main drivers of aligning human and mouse ductal groups. On the other hand, the alpha cell population had a high value of inter-species similarity (0.62) owing to top positive-influence genes, e.g., *GCG* and *TTR* (Fig. 4F).

**Fig. 5:**
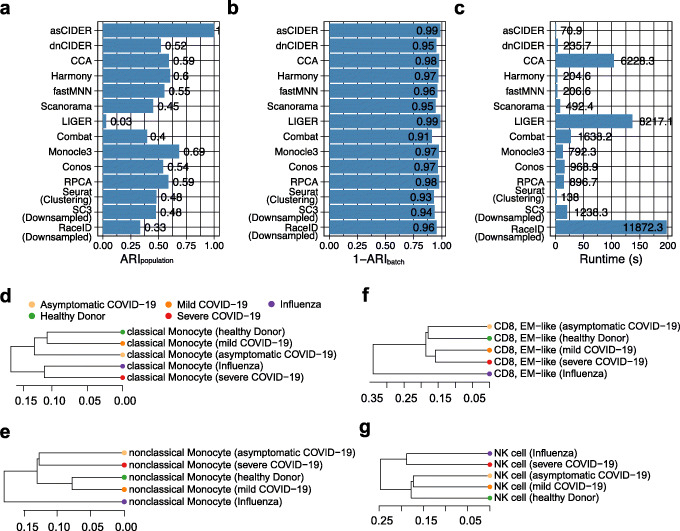
CIDER identified cross-condition cell classes in the PBMC data from healthy donors and patients with COVID-19 or influenza (Dataset 5). **A**–**C** The distribution of ARI_population_ (**A**), 1-ARI_batch_ (**B**), and runtime (**C**) of benchmarked algorithms. SC3 and RaceID were ran on the dataset downsampled by the factor of 5. **D**–**G** Dendrograms show the local relationships of the classical monocyte population (**D**), nonclassical monocyte population (**E**), the EM-like CD8 T cell population (**F**), and the natural killer (NK) population (**G**) from donors with different health conditions

We next tested the capability of coping condition effects on data from a recent COVID-19 study (Dataset 5) [22]. This dataset contained 59,572 PBMCs collected from healthy donors, patients with severe influenza, and patients with various severity of COVID-19 (asymptomatic, mild, and severe). These cells were cataloged into 15 populations: lgG− B cell, lgG+ B cell, effector memory (EM)-like CD4+ T cell, non-EM-like CD4+ T cell, EM-like CD8+ T cell, non-EM-like CD8+ T cell, NK cell, classical monocyte, intermediate monocyte, nonclassical monocyte, dendritic cell (DC), uncategorized 1, uncategorized 2, red blood cell (RBC), and platelet. For this dataset, the health condition was treated as the confounding factor for correction. Among the benchmarked methods, asCIDER had the highest ARI_population_, while the other methods, except LIGER, Combat, RaceID, and Monocle3, had similar ARI_population_ values between 0.45 and 0.60 (Fig. 5A). The overall low level of ARI_population_ was likely due to the manually curated and merged cell annotations [22], where the similarity between defined cell populations might not reflect the statistical similarity defined by these clustering and integration algorithms. Besides, because the cell type annotations were generated from CCA-corrected data, it was expected that the comparison results favored CCA and similar methodologies. The lower ARI_batch_ values of Seurat and SC3 clustering results suggested that this dataset was mildly confounded by the effect of health conditions (Fig. 5B). AsCIDER consumed the shortest running time, while dnCIDER was slightly slower than Harmony and fastMNN but faster than other integration methods (Fig. 5C). After regressing out the systematic effect of health conditions, the inter-group distance matrix generated by asCIDER unraveled the cell-type-specific local relationship of various conditions. For example, the populations of classical monocytes, natural killer (NK) cells, red blood cells (RBC), dendritic cells (DC), and lgG+ B cells from patients with severe COVID-19 were more akin to the ones from patients with severe influenza than the ones from patients with mild or asymptomatic COVID-19, while nonclassical monocytes and effector memory (EM)-like CD8 T cells were not (Fig. 5D–G, Additional file 1: Fig. S6A-C).

**Fig. 6:**
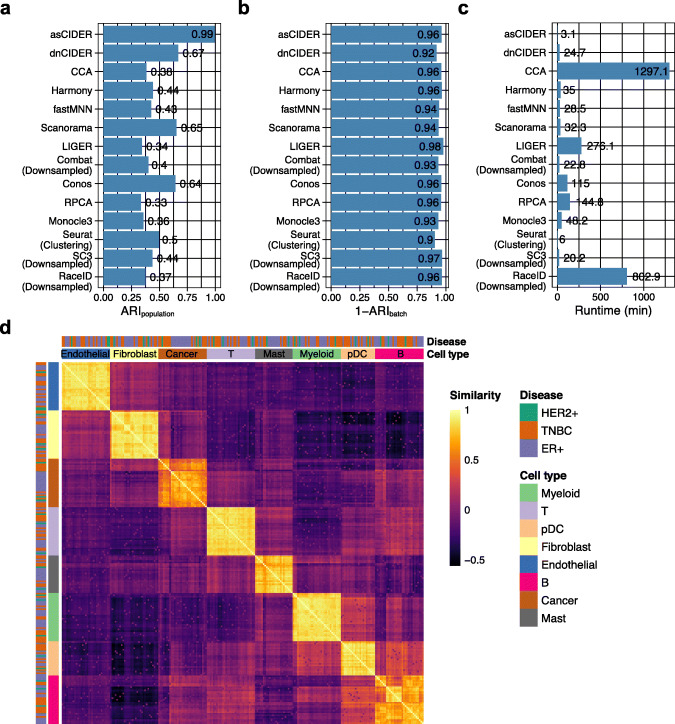
CIDER identifies cross-donor populations in the breast tumor data of 170,350 cells from 31 patients (Dataset 6). **A**–**C** The distribution of ARI_population_ (**A**), 1-ARI_batch_ (**B**), and runtime (**C**) of benchmarked algorithms. **D** Heatmap shows the inter-group similarity matrix for donor-specific initial clusters of asCIDER. Tiles are colored by the similarity levels. The annotation bars are colored by diseases or cell types

To demonstrate the scalability of CIDER, we benchmarked CIDER and other methods on a breast cancer dataset (Dataset 6) containing 170,350 cells from 31 patients with the estrogen receptor-positive (ER+) subtype, the human epidermal growth factor receptor 2-negative (HER2−) subtype, and the triple-negative breast cancer (TNBC) [23]. For each patient, two samples were collected, one before the treatment and one during the subsequent surgery. Thus, three potential covariates existed, namely the donor effect, the treatment effect, and the disease effect, and donor was the nesting variable to disease. To identify the cross-patient populations, we generated patient-specific initial clusters and then used donor and treatment (pre-treatment or on-treatment) as covariates to calculate the IDER-based similarity matrix, which enabled regressing out donor and treatment effects. Compared to other methods, CIDER methods had higher accuracy in identifying cross-donor populations (Fig. 6A). They were also less affected by the donor effect compared to solely using Louvain clustering (Fig. 6B). Both algorithmic variants dnCIDER and asCIDER consumed less time than other integration methods applied to the full dataset (Fig. 6C). Other than providing the clustering results, asCIDER also revealed that the tumor cells and, interestingly, the B cells had higher levels of intra-population heterogeneity, even after regressing out the systematic cross-population donor and treatment effects (Fig. 6D). Such heterogeneity was expected in the tumor cells [23], while the one in B cells has remained obscure.

Overall, it suggested that the clustering performance of asCIDER and dnCIDER was more accurate on data confounded by technical effects, species difference, disease variability, and inter-donor variability, compared to the clustering results generated from the corrected low-dimensional representations. CIDER methods could also provide insights into the intra-population heterogeneity across different conditions.

### CIDER as a ground-truth-free test metric of integration

**Fig. 7:**
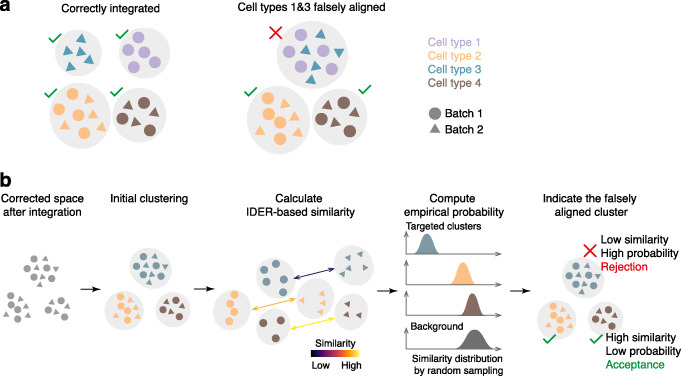
CIDER can evaluate the biological accuracy of integration results without reliance on ground truth. **A** The diagram shows two scenarios, one, where the cell types from two batches are correctly aligned, and one, where the two heterogeneous cell types (1 and 3) from two batches are falsely aligned, often because of overcorrection. **B** The schematic diagram elucidates the workflow for assessing the integrated outcome to identify falsely aligned clusters, which have lower inter-group similarity. CIDER can identify the second scenario without prior information of cell types

One of the common pitfalls of multiple dataset integration is incorrect alignment, where two heterogeneous groups of cells are merged in the corrected space (Fig. 7A). Although existing test metrics, such as the cell-type local inverse Simpson Index (cLISI) [11], can measure the local impurity in the joint low-dimensional representation, its major limitation is the demand for predefined cell populations. To address this limitation, we embedded CIDER into a workflow of evaluating the integration outcome, and our evaluation method does not require the ground truth of cell type annotations (Fig. 7B). In this workflow, after data are corrected by a chosen integration tool, an initial clustering step generates cross-batch clusters based on the corrected expression matrix or low-dimensional representation. Using the IDER metric, the inter-group similarity is calculated between the initial clusters split by batches. The empirical probability of rejecting the alignment is next computed by comparing the distributions of similarity between the targeted cluster and the background. Low similarity or a high empirical probability putatively indicates the falsely aligned cluster, i.e., rejection of the fact that cells from a cross-batch cluster belong to a homogeneous population.

**Fig. 8:**
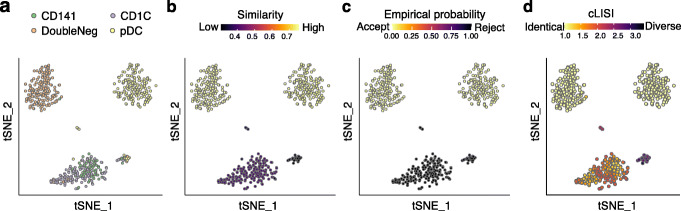
CIDER identifies the falsely aligned CD1C and CD141 subtypes in the dendritic cell data (Dataset 7). **A**–**D** *t*-SNE plots of the CCA-corrected data, where cells are colored by cell populations (**A**), the similarity score computed by CIDER (**B**), the empirical probability of rejection computed from the background distribution (**C**), and the cLISI values (**D**)

We applied CCA on the dendritic cell dataset (Dataset 7) [24], which contains four cell subtypes (CD141, CD1C, double negative [DoubleNeg], and plasmacytoid dendritic cell [pDC]). The integration algorithm is prone to merging the CD141 cell population and the CD1C population incorrectly (Fig. 8A) [15]. After integration and dimensionality reduction, we applied CIDER on the corrected low-dimensional representation to compute the similarity and empirical probabilities (Fig. 7B and Additional file 1: Fig. S7A-D). The cluster that had lower similarity and high probability of rejection was the mixture of the CD141 and CD1C populations, while the other two clusters (DoubleNeg and pDC) with high similarity and low empirical probability were properly aligned (Fig. 8B, C). It demonstrated that CIDER could accurately identify falsely aligned populations. To further visualize the local diversity and compare it with the CIDER metric, we used the cLISI metric [11], where the cLISI over 1 indicated the local heterogeneity of cell classes. The results of CIDER were in accord with cLISI (Fig. 8D).

**Fig. 9:**
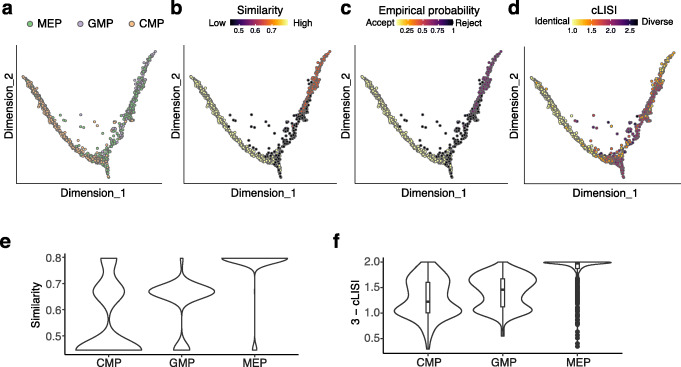
CIDER identifies a high level of heterogeneity within the CMP population in the mouse hematopoietic progenitor data (Dataset 8). **A**–**D** Diffusion maps of the CCA-corrected data, where cells are colored by cell populations (**A**), the similarity score computed by CIDER (**B**), the empirical probability of rejection (**C**), and the local purity represented by 3-cLISI (**D**). **E** The distribution of agreement (*y*-axis), denoted by the similarity calculated by CIDER, between two experiments for three cell populations (*x*-axis), CMP, GMP, and MEP. **F** The distribution of agreement, denoted by 3-cLISI, between two experiments

We next tested CIDER on the mouse hematopoietic progenitor data (Dataset 8) with the continuous data structure [25, 26]. Cells of this dataset were assigned to three populations, the common myeloid progenitor (CMP), the megakaryocyte/erythrocyte progenitor (MEP), and the granulocyte/macrophage progenitor (GMP), and profiled by two platforms, MARS-seq [26] and Smart-Seq2 [25] (Fig. 9A). After integration and dimensionality reduction, we used CIDER to compute the similarity and empirical probabilities. The CIDER metrics indicated that the cells around the bifurcating point shared lower levels of agreement between the two experiments (Fig. 9B, C). Based on the ground truth of cell annotations, the results of cLISI also suggested that multiple populations were mixed around the bifurcating point (cLISI ≥ 2; Fig. 9D). Moreover, the results of CIDER showed that the alignment scores of CMP, the direct ancestor of both MEP and GMP, were lower than those of MEP and GMP between two experiments, which was consistent with the distribution of 3-cLISI (Fig. 9E, F). This is likely due to the higher level of heterogeneity in the predefined CMP population compared to MEP [26]. Taken together, we demonstrated that CIDER could accurately evaluate the local biological homogeneity without relying on predefined cell annotations.

## Discussion

In this work, we presented a meta-clustering framework, CIDER, for scRNA-Seq data integration and evaluation. The benchmarking demonstrated the performance of CIDER regarding the accuracy of recognizing cellular populations, the effectiveness of removing batch effects, and its scalability.

CIDER used a novel and intuitive strategy that measures the similarity by performing group-level calculations, which stabilize the gene-wise variability. Compared to other distance measures or anchors used for clustering and integration [6, 9], we show that IDER is versatile in its ability to quantify biological similarity and readily interpretable. CIDER can be exploited for preliminary analysis, standalone clustering, or independent validation. Since IDER is built on a different rationale from conventional integration approaches, the similarity graph it generates can provide insights that can be treated as an alternative to standard techniques, which often cannot genuinely preserve long-distance and short-distance relationships. Moreover, CIDER can compute a similarity score between cell groups from two conditions, enabling the inference of local relationships based on the expression profiles. Among other methods, Scanorama [8] can also calculate an alignment score for pairs of datasets for better interpretability, but it is derived from the membership of shared nearest neighbors rather than directly estimated from expression profiles.

A common question of integration is which effects should be considered. Two criteria, the magnitude of the bias and their relevance to the purpose of the study, can be used to choose covariates for correction. In the first scenario, such as Dataset 2 (simulated data) and Datasets 3 (PBMC), the simulated batch effect and the technical effect introduced bias into the clustering if not corrected (Figs. 2 and 3C and A), indicating the covariates for regression. On the other hand, advances in the droplet-based scRNA-Seq platform and the cryopreservation technique have enabled the minimization of technically introduced batch effects. Thus, in the experiments that follow one consistent experimental protocol and include multiple donors, the inter-sample variability can be largely attributed to the “biological” variability, such as donors’ condition and genetic diversity [27, 28]. In this scenario, the selection of covariates for regression can be based on the relevance to the research goal. For example, the health condition in Dataset 5 (COVID-19 versus severe flu) and the donor, as well as the treatment, in Dataset 6 (breast cancer) were corrected to identify cross-condition and cross-donor populations.

Multiple sample integration has become one of the most frequently used tools for scRNA-Seq data analysis [29]. Along with the rapidly growing amount of available scRNA-Seq data, the recent advances in neural network models and approaches for transfer learning have facilitated the query-reference mapping [30]. This highlights the importance of accurate integration. We demonstrated the usefulness of CIDER for evaluating the integration outcome, which can be used to select integration tools and tune the parameters if a joint low-dimensional representation is desired.

CIDER is currently designed for scRNA-Seq data and cannot be used for the integration of single-cell multi-modal data [31, 32]. Future work can be focused on adapting the linear model embedded in CIDER for this purpose. Although the group-level analysis CIDER performs is coarse-grained, CIDER can be applied to data with continuous structures, as we demonstrated; further work to develop specific extensions in this methodological direction is required.

## Conclusions

CIDER provides a clustering framework for integrative analysis of multiple scRNA-Seq datasets, enabling identifying cell assignments across datasets and validating the integration output for the assembly of multiple scRNA-Seq datasets.

## Methods

### Measurement of inter-group similarity

The infrastructure of IDER was built on limma-trend [17] or voom [33]. Both limma-trend and voom estimate the mean-variance relationship non-parametrically by locally weighted regression and then leverage the estimation for DE analysis. The difference between limma-trend and voom is that the mean-variance relationships exploited by them are at the gene level and at the level of individual observations, respectively.

Limma methods were selected out of a collection of tools for DE analysis. First, limma-trend and voom were top performers for scRNA-Seq data demonstrated by a recent benchmarking study [18]. Secondly, the linear models of limma enabled complex design. Additionally, we benchmarked limma with other top performers (MAST [34] and edgeR [35]) in a simulated dataset confounded by batch effects. MAST uses a hurdle model of a two-part generalized linear model, aiming to model the bimodality expression pattern of zero-inflated scRNA-Seq data, while edgeR fits the coefficients and the dispersion parameters using the negative binomial distribution. In our benchmarking experiment, the limma methods detected the signal-to-noise better than MAST and edgeR, and its computing speed was much faster (Additional file 1: Fig. S1A, B), which was consistent with previous results [33]. Moreover, limma-trend was faster than voom, because voom has an additional step of inferring variance at the level of individual observations. Limma-trend was recommended when the runtime is a major concern, while voom may perform slightly better when library sizes are unequal [33].

IDER is aimed to measure inter-group similarity. In the scenario of multiple batches, IDER first compares two groups, *g*_*i*_ and *g*_*j*_*’*, with the background, i.e., cells that do not belong to *g*_*i*_ and *g*_*j*_*’*, respectively (Fig. 1A). For each comparison, the DE analysis is performed with the linear regression including covariates of group (*g*_*i*_, *g*_*j*_*’*, and background), batch, and scaled cellular detection rate. The cellular detection rate measures the number of genes detected per cell as previously described [34]. After the estimated coefficients are computed, the DE signature, vector *d*_*i*_, for group *g*_*i*_ (or *d*_*j*_*’* for group *g*_*j*_*’*) is computed by fitting the contrast of *g*_*i*_ – background (or *g*_*j*_*’* – background). The length of *d*_*i*_ or *d*_*j*_*’* is equal to the number of genes used. The DE signature is denoted by the estimated coefficients, i.e., log_2_ fold-change. Between the two groups, *g*_*i*_ and *g*_*j*_*’*, the similarity *s(g*_*i*_*, g*_*j*_*’)* is measured by the Pearson correlation coefficients between DE signatures, *d*_*i*_ and *d*_*j*_*’*. This similarity measure ranges from −1 to 1. IDER can also be used to measure inter-group similarity for data with multiple levels of confounding factors. Under this circumstance, the additional covariates were included as a covariate in the regression model. For example, in the breast cancer data (Dataset 6), both the donor effect and the treatment effect were included in the regression model ${E}_{ij k}={C}_{j,{z}_{ik}}+{B}_{jk}+{T}_{ij}+{R}_i$, where *E*_*ijk*_ denotes the expression level of gene *j* in cell *i* of donor *k*, *z*_*ik*_ cell assignment of cells of cell *i*, *C* cluster effect, *B* donor effect, *T* treatment effect, and *R* cellular detection rate.

### CIDER for identifying cell populations

To cluster multi-batch data, CIDER consists of three steps: initial clustering, computing the similarity matrix, and final clustering. For dnCIDER, we first used Louvain clustering to cluster cells within each batch. Pairs of batch-specific clusters with high similarity of IDER were merged, generating the initial clusters for the next step. For asCIDER, we concatenated the batch tag and the cell annotation as the initial cluster. Next, to generate the similarity matrix, the pairwise similarity was computed for inter-batch initial clusters by IDER. We downsampled each initial cluster to the same size (35 to 50 cells). We do not suggest downsampling to a number smaller than 15. For initial clusters smaller than this size, we allowed replacement for sampling. To visualize the similarity among initial clusters, this similarity matrix was transferred to a graph by using igraph in R (https://igraph.org/r/). In the final clustering step, the similarity matrix ***S*** was transferred to a distance matrix by 1 − ***S*** and the initial clusters were merged by the hierarchical agglomerative clustering with complete linkage, enabling the initial clusters with the highest similarity to be merged first. For large datasets, parallel computation (R package doParallel) was used to shorten the runtime.

#### Interpretability

To measure the influence of individual genes on the inter-group similarity, the correlation *r*_*i*_ of only leaving gene *i* out was calculated and the Fisher *z*-transformation $\frac{1}{2}\mathit{\ln}\left(\frac{1+{r}_i}{1-{r}_i}\right)$ was taken, which transformed the sample distribution of the correlation coefficients to the Gaussian distribution. The influence was computed as $\frac{1}{2}\mathit{\ln}\left(\frac{1+r}{1-r}\right)-\frac{1}{2}\mathit{\ln}\left(\frac{1+{r}_i}{1-{r}_i}\right)$, where *r* denotes the correlation including all genes.

#### Downstream analysis of marker genes

We used limma-voom to identify the marker genes. For Dataset 3, clustering results, batch information, and the cellular detection rate were used to construct the design matrix. The linear model was first fitted for the given design matrix, and then the estimated coefficients were computed for the contrasts between the target cluster and the background. Empirical Bayes statistics were calculated. Expression of the top marker genes with Benjamini-Hochberg-adjusted *p* values lower than 1.83 × 10^−18^ and log_2_ fold-changes over 1.47 were visualized using the function DotPlot from Seurat.

### External data

#### Cell line data (Dataset 1) [19]

We obtained the data of 293T cells and Jurkat cells from http://scanorama.csail.mit.edu/data.tar.gz [8]. This dataset came from three batches. The first batch has only 293T cells, the second batch only Jurkat cells, and the third batch 1:1 mixture of these two cell lines.

#### Human PBMC data (Dataset 3) [19]

This dataset contains 14,876 cells of human PBMC samples from two platforms (10x 3’ and 10x 5’). The raw count matrix and the sample information were downloaded from https://hub.docker.com/r/jinmiaochenlab/batch-effect-removal-benchmarking, which were curated in the recent benchmarking study [15]. Cells were annotated [20]. Cells with at least 500 genes detected were kept for further analysis. Putative doublets were filtered by DoubletFinder [36] for each batch. The first 10 PCs were used for clustering analysis. The resolution of Louvain clustering was 0.4.

#### Cross-species pancreatic data (Dataset 4) [21]

The count matrix and sample information were downloaded from NCBI GEO accession GSE84133. We kept cells with minimum 500 genes detected for downstream analysis. Doublets were filtered by DoubletFinder. The gene set shared by both the human and the mouse was kept for downstream analysis. The human gene *INS* was treated as the mouse gene *Ins1* as previously described [9].

#### COVID-19 data (Dataset 5) [22]

The 10x data were downloaded from GSE149689, and the cell annotations were downloaded from https://junglab.wixsite.com/home/db-link.

#### Breast cancer data (Dataset 6) [23]

The count matrix and cell annotations were downloaded from https://lambrechtslab.sites.vib.be/en/single-cell. Cells were cataloged into eight cell types, namely cancer cell, myeloid, T cell, pDC, fibroblast, endothelial, B cell, and mast. Putative doublets were filtered by DoubletFinder [36] for each batch.

#### Human dendritic data (Dataset 7) [24]

The data were downloaded from https://hub.docker.com/r/jinmiaochenlab/batch-effect-removal-benchmarking [15]. The data contained four cell subtypes (CD141, CD1C, DoubleNeg, and pDC) from two batches [24]. The raw count matrix and the sample information were also downloaded from the curated set [15]. Cells with less than 500 genes detected were removed.

#### Mouse hematopoietic progenitor data (Dataset 8) [25, 26]

The data were downloaded from the curated set [15] and contain three cell populations, named CMP, GMP, and MEP, sequenced by two platforms (MARS-seq and Smart-seq). CMP was recognized as the direct ancestor of GMP and MEP.

### Integration pipelines

#### Seurat CCA and RPCA

We used the recommended CCA and RPCA correction pipelines of Seurat v4.0.3 [9]. We first split objects by batches, followed by normalization and selection of HVGs based on the relationship between mean and variance. The integration anchors were identified to integrate the data. The corrected low-dimensional representation was used for Louvain clustering.

#### fastMNN

We used scran v1.14.5 to identify HVGs, which were used as the input of fastMNN (R package batchelor v1.2.4) [6]. The fastMNN-corrected low-dimensional representation was used for Louvain clustering.

#### Scanorama

We used Scanorama [8] via reticulate v1.16 in R as suggested by the Scanorama repository (https://github.com/brianhie/scanorama). The corrected embedding was used for Louvain clustering.

#### Harmony

We used the RunHarmony function of Harmony v1.0 [11] to perform integration and used the first 15 corrected PCs as the input of Louvain clustering with the resolution of 0.4.

#### LIGER

We used rliger v1.0.0 [12]. The Seurat object was first converted to the Liger object, followed by normalization, HVG selection, scaling, integrative non-negative matrix factorization, construction of the shared factor neighborhood graph, and the Louvain clustering.

#### Combat

We used R package sva v3.34.0 [13]. The count matrix was log_2_ transformed and corrected by the Combat function. The corrected expression matrix was used as the scaled data for the HVG selection, PCA computing, and Louvain clustering. The downsampling factor of Dataset 6 was 3.

#### Conos

R packages conos v1.4.2 [14], SeuratWrappers v0.3.0, and pagoda2 v1.0.5 were used. The data were first split by the batch variable and preprocessed by the Seurat pipeline. The joint graph was built in the PCA space, and then, the cell clusters were identified as communities in the joint graph.

#### Monocle3

We used monocle3 v1.0.0 [10] for preprocessing, dimension reduction, batch effect removal [6], and clustering [37].

### Clustering pipelines

#### Seurat Louvain clustering

We used the suggested pipeline of Seurat v3.1.5 [5]. The top 2000 HVGs were used to compute PCs, while the first fourteen PCs were used for Louvain clustering with the resolution of 0.4.

#### SC3

We used SC3 v1.14.0 [3]. The number of clusters based on ground truth was given to the clustering function. The downsampling factor of Datasets 5 and 6 was five.

#### RaceID

We used the suggested pipeline of RaceID v0.1.9 [4], including filterdata, getfdata, compdist, and clustexp. The number of clusters based on ground truth was given to the clustering function. As the SingleCellExperiment object that SC3 and RaceID depended on consumed a substantial amount of memory, the data were downsampled for Datasets 5 and 6 before applying SC3 and RaceID. The downsampling factor of Datasets 5 and 6 was five.

### Proof-of-concept analysis

The cell line dataset (Dataset 1) was corrected by Scanorama as previously described [8]. The first two components of *t*-SNE were used to perform Hierarchical DBSCAN (R package dbscan v1.1) with the minimum size of clusters set at 75. The output of DBSCAN and the batch information were combined to generate initial clusters. The Scanorama correction was used here as the ground truth, as its correctness has been demonstrated previously [8]. The initial clusters were downsampled to the size of 50 cells. The IDER-based similarity matrix was computed among the initial clusters to demonstrate the ability to capture biological variance.

### Data simulation

We used Splatter v1.10.0 [38] to simulate scRNA-Seq data. We first simulated a dataset with five groups and three batches and removed groups 4 and 5 from batch 1, groups 1 and 5 from batch 2, and groups 1 and 3 from batch 3. This generated the non-overlapped scenario (Dataset 2). The replications were generated in the same way with various seed values.

### Benchmarking clustering performance

The adjusted Rand index (ARI) was used to measure the consistency between clustering results and ground truth.

#### ARI_population_

We calculated the ARI between clustering results and the annotation of cell populations, termed ARI_population_. It indicates the accuracy of identifying cell populations.

#### 1-ARI_batch_

We also computed the ARI between clustering outcome and the annotation of batches, termed ARI_batch_. It represents the confounding effects of batches. Therefore, a larger value of 1-ARI_batch_ indicates that the clustering result is less confounded by batch effects.

#### Runtime

The runtime was tested on a Linux server with a maximum number of cores of 16. Given that CIDER needed to compute pairwise similarity, the runtime of CIDER was approximately *O*(*n*^2^), where *n* denotes the number of batch-specific initial clusters. It was also positively associated with the covariates and the number of genes included in the regression.

### CIDER for evaluating integration

In this evaluation workflow, the batch-corrected low-dimensional representation was first used to partition cells into multi-batch clusters. These multi-batch clusters were further divided into batch-specific subclusters. Within each cluster, the inter-group similarity was calculated between subclusters from a pair of batches, while the batch effects were regressed out by using the IDER metric. Higher levels of inter-group similarity indicated better quality of integration for the cluster. For two batch-specific subclusters from the same cluster, we could estimate the probability of whether they come from a true biological population (either cell type, subtype, or state). To estimate the probability, we assumed that the two mutual nearest batch-specific groups with the highest similarity are from the same population (“mutual nearest neighbor” hypothesis) and that the variability within a given biological population is at an almost constant level (“constant variability” hypothesis). By further partitioning the combination of these two batch-specific subclusters, we could get a distribution of the variability within this merged cluster. An empirical probability was next calculated for each pair of subclusters from the same cluster to indicate the probability of belonging to the same population.

The cLISI metric, computed by R package lisi [11], was used to validate the similarity and the empirical probability calculated by CIDER. LISI measures the population diversity within the neighbors of a given cell, and the neighborhoods are defined by Gaussian kernel-based distributions. Here, cLISI was calculated as the LISI between the ground-truth annotations of cell populations in the batch-corrected *t*-SNE space.

## Supplementary Information

**Additional file 1: Fig. S1-S7 and Table S1.**

**Additional file 2.** Review history.

## Data Availability

Raw data are available from SRA under accession number SRP073767 [19]; GEO under accession numbers GSE84133 [21], GSE149689 [22], GSE94820 [24], GSE81682 [25], and GSE72857 [26]; and the European Genome-phenome Archive (EGA) under accession number EGAD00001006608 [23]. Analysis scripts of this work are deposited on GitHub https://github.com/zhiyhu/CIDER-paper [39] and Zenodo https://zenodo.org/record/5715956 [40]. The R package CIDER is available at GitHub (https://github.com/zhiyhu/CIDER) [41] and Zenodo https://zenodo.org/record/5716025 [42]. The R package CIDER has also been deposited at CRAN (https://CRAN.R-project.org/package=CIDER).
