# Supplementary material for: CIDER: an interpretable meta-clustering framework for single-cell RNA-seq data integration and evaluation
Source: Genome Biol. 2021 Dec 13;22:337. doi: 10.1186/s13059-021-02561-2 (PMC8667531; doi:10.1186/s13059-021-02561-2)
Supplement: Supplementary file 1 — Additional file 1: Fig. S1-S7 and Table S1. [file 13059_2021_2561_MOESM1_ESM.docx]

**
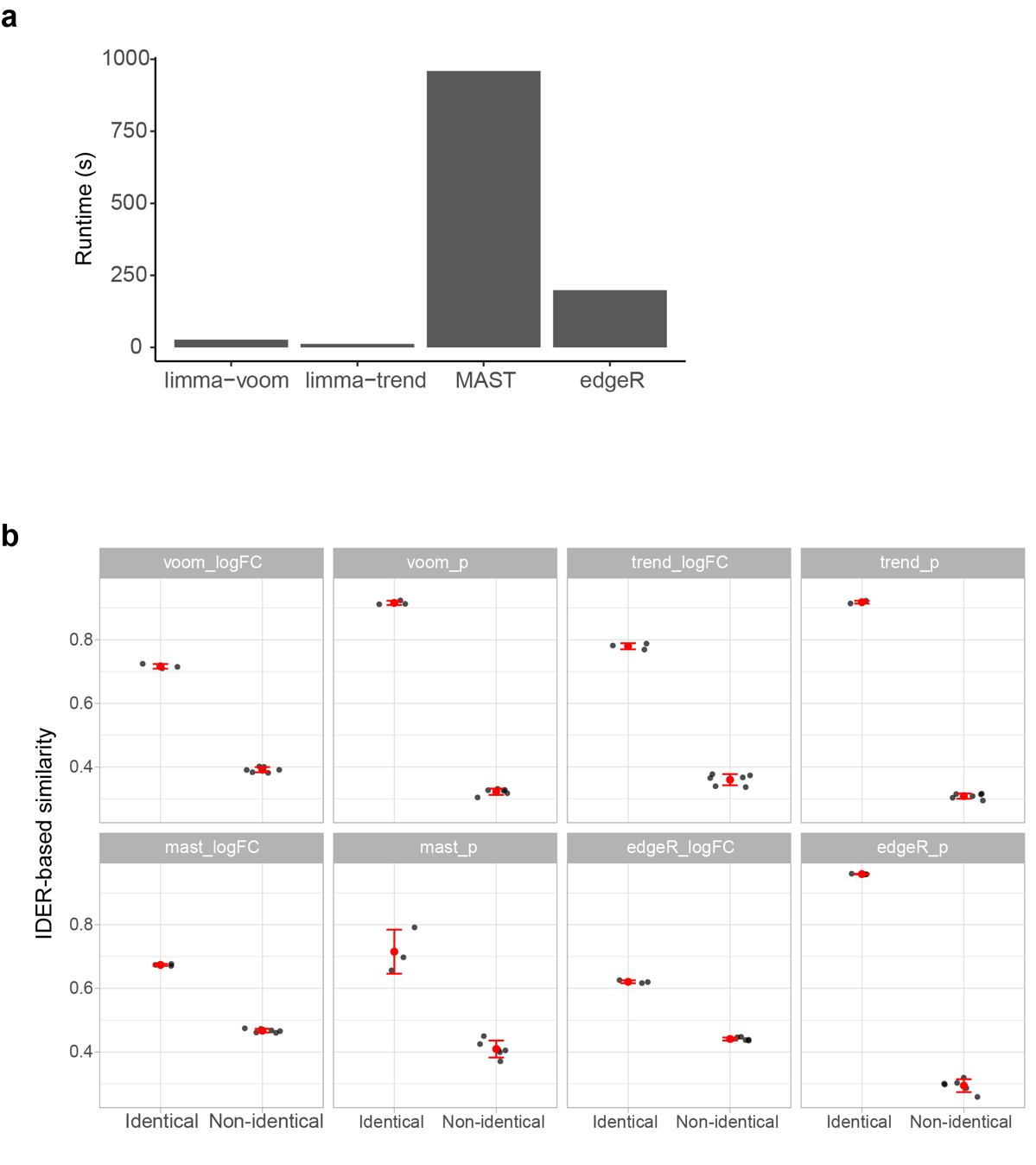
**

**Fig S1. Benchmarking limma methods with MAST and edgeR for differential expression analysis.**

**(a)** Runtime of four methods used to compute the IDER matrix of a simulated dataset with two batches and 300 cells per batch. The computing speed of limma methods is faster than MAST and edgeR. Furthermore, limma-trend is faster than voom, because voom calculates observation-level variance in addition.

**(b)** Distribution of similarity levels computed by different methods. The signal-to-noise ratios detected by limma methods are better than edgeR and MAST. The x-axis shows whether the group pairs come from identical population or non-identical populations. The y-axis denotes the IDER-based similarity between a pair of groups. Each dot is a group pair. The large red dot indicates the median and error bars show standard deviations.

**
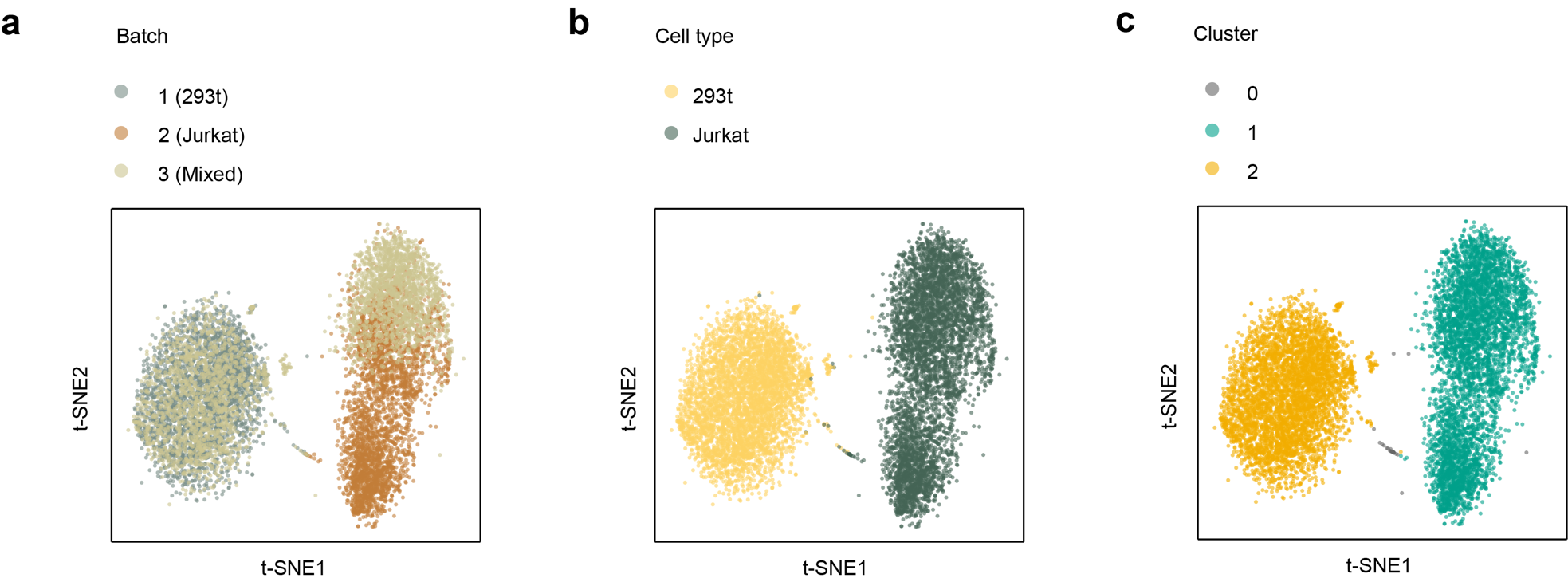
**

**Fig S2. Compositions of the cell line dataset (Dataset 1).** *t*-SNE plots of the Scanorama-corrected data. Each dot denotes a cell, colored by batches (a) and cell types (b).

**
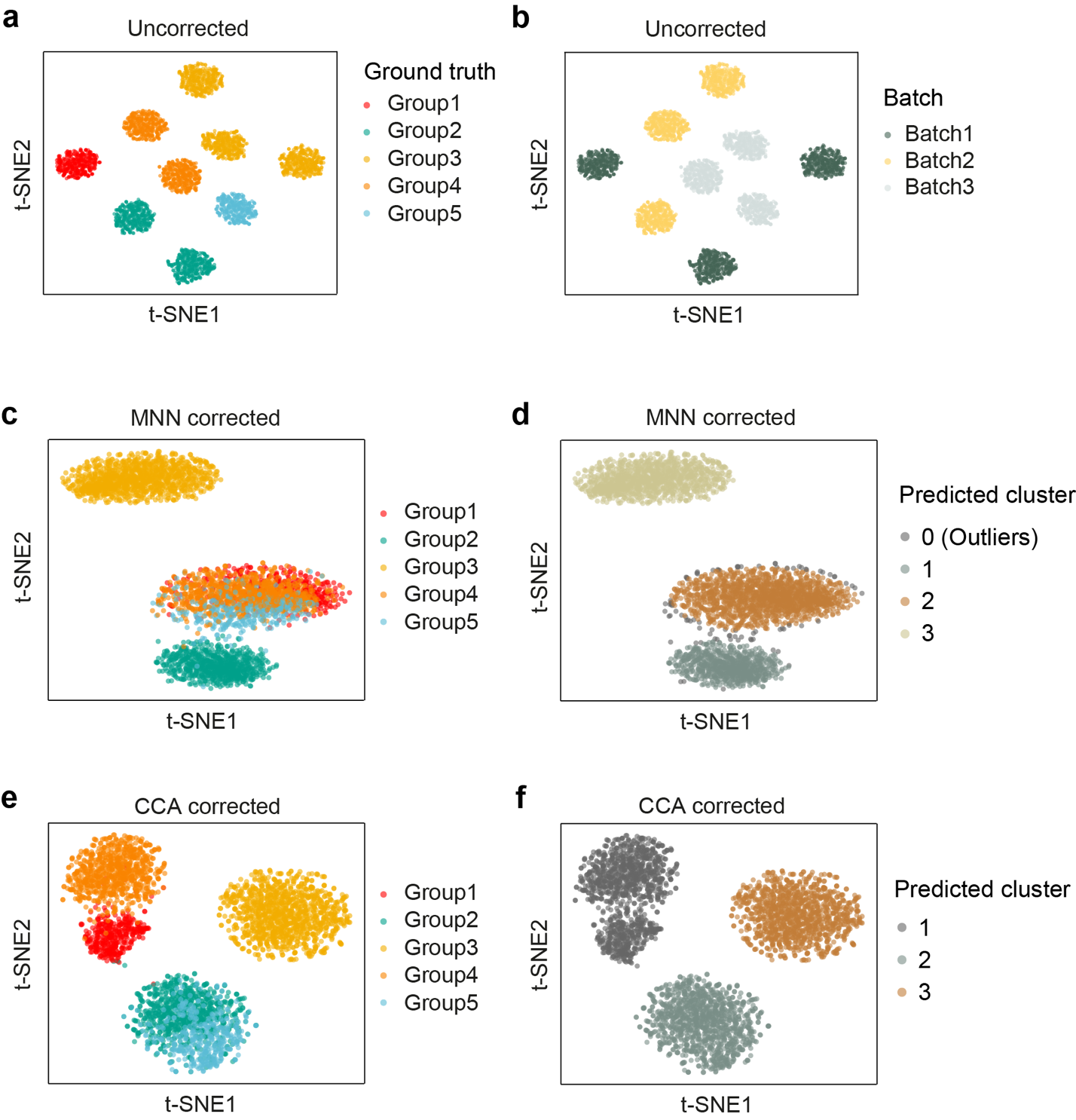
**

**Fig S3. Compositions of the simulation dataset (Dataset 2). (a-b)** *t-*SNE plots of uncorrected data, where cells are colored by populations (a) and batches (b). **(c-d)** *t*-SNE plots of MNN-corrected data. Cells are colored by populations (c) and by clustering results of DBSCAN (d). **(e-f)** *t*-SNE plots of CCA-corrected data. Cells are colored by populations (e) and by clustering results of DBSCAN (f).

**
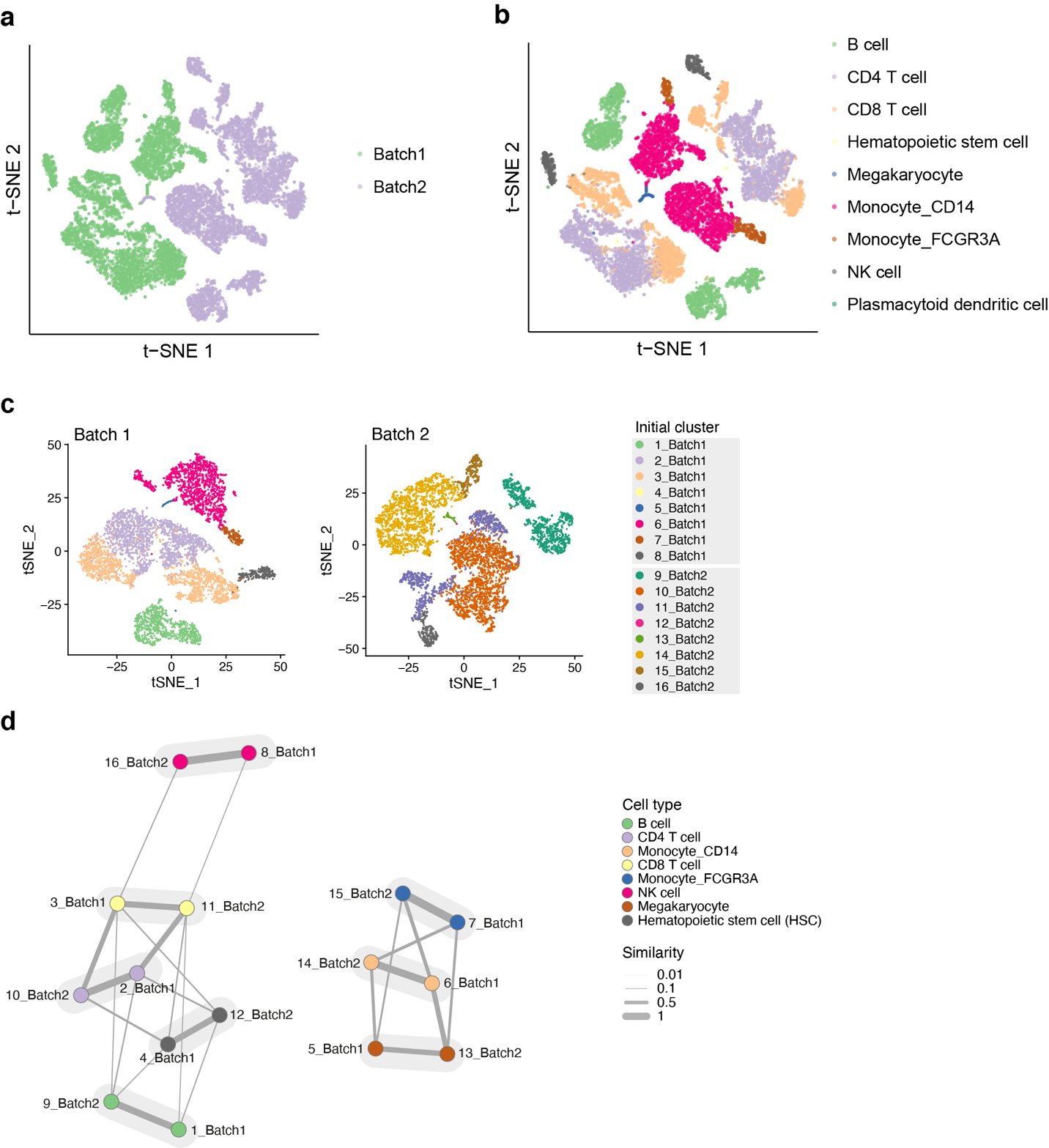
**

**Fig S4. Benchmarking on the human PBMC data (Dataset 3). (a-b)** *t*-SNE plots of uncorrected Dataset 3. Cells are colored by batches (a) and populations (b). **(c)** *t*-SNE plots of Batches 1 and 2. Cells are colored by asCIDER initial clusters. **(d)** Network graph shows the inter-group similarity among initial clusters of asCIDER. Vertexes represent initial clusters, colored by populations. The width of edges represents the similarity levels. Grey circles denote the initial clusters that are merged in the final clustering step.


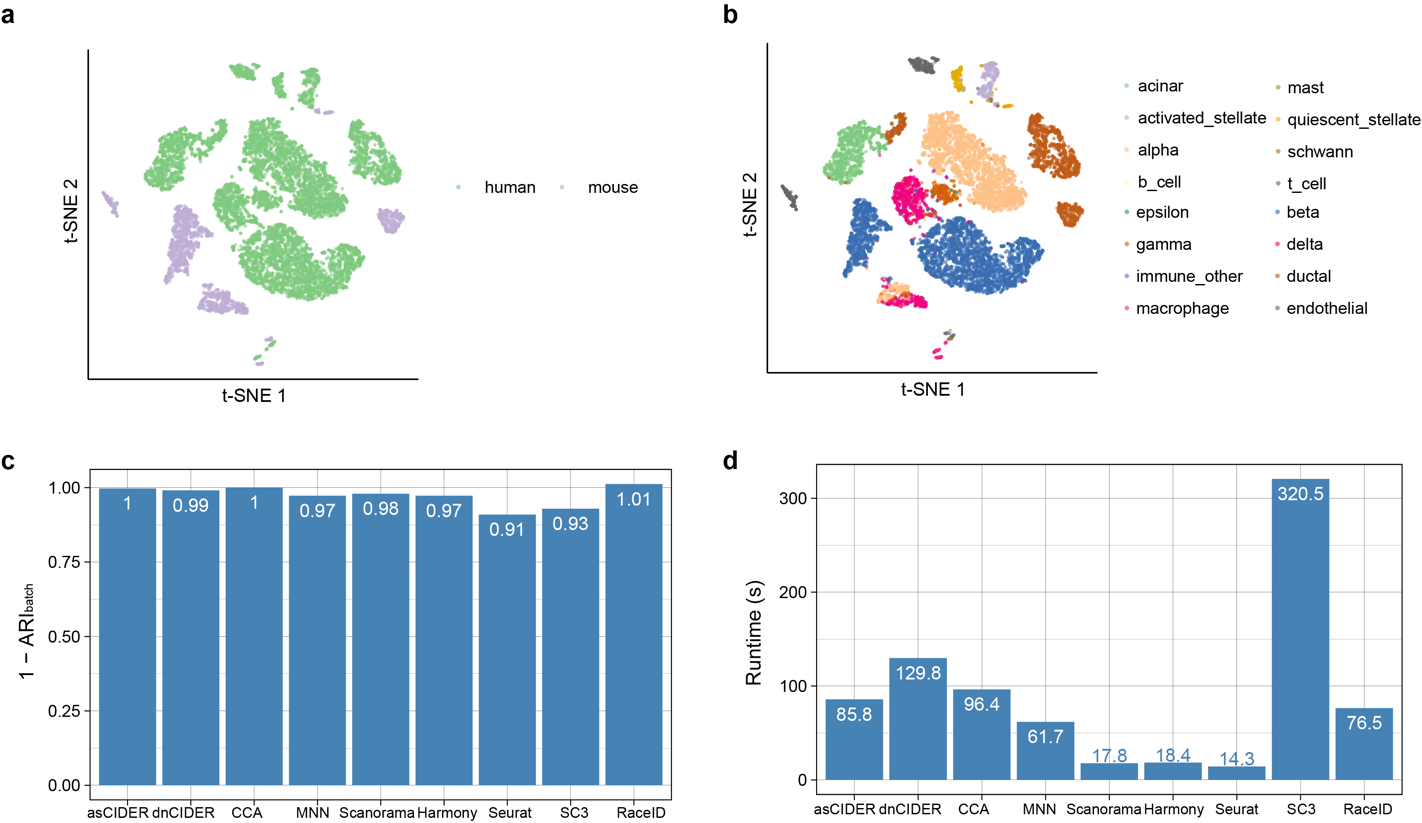


**Fig S5. Benchmarking on the cross-species pancreas data (Dataset 4). (a-b)** *t*-SNE plots of uncorrected data, where cells are colored by batches (a) and populations (b).


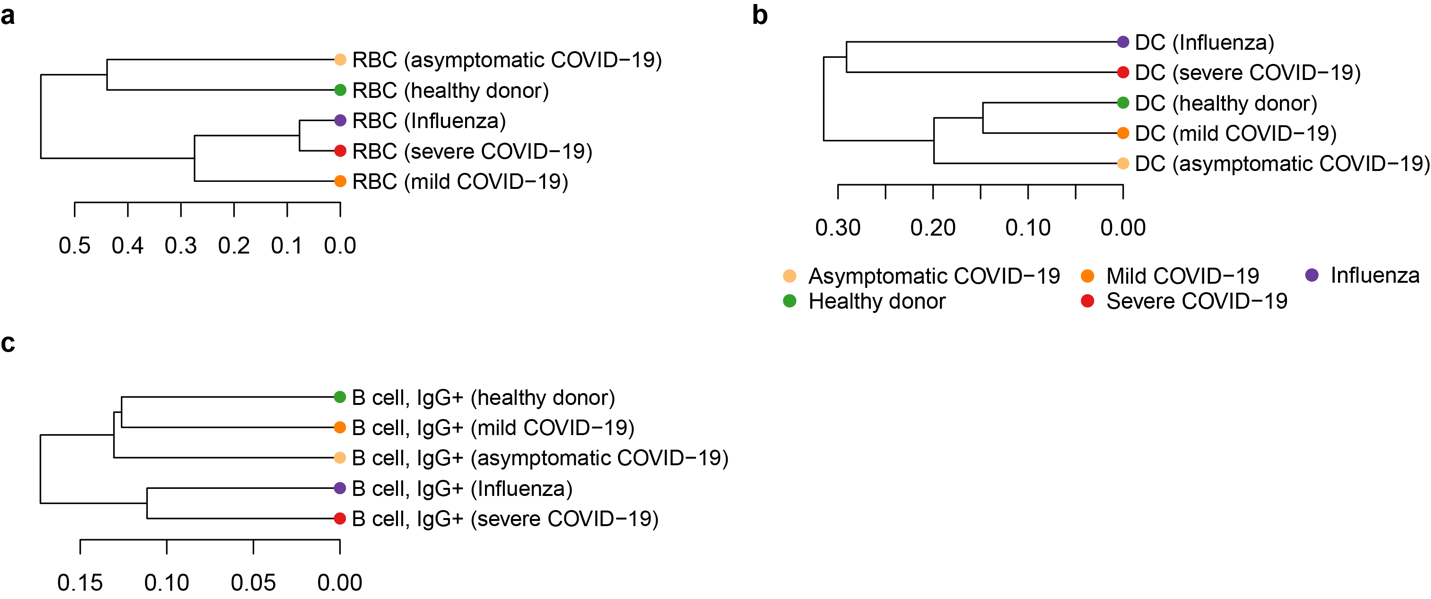


**Fig S6. Benchmarking on the human PBMC data from healthy donors and patients with asymptomatic COVID-19, mild COVID-19, severe COVID-19 or severe Influenza (Dataset 5). (a-c)** Dendrograms show the local relationships of the red blood cell (RBC) population (a), the dendritic cell (DC) population (b) and the lgG+ B cell population (c). Each leaf represents a cell population from one group of donors. The x-axis shows the height of branching.


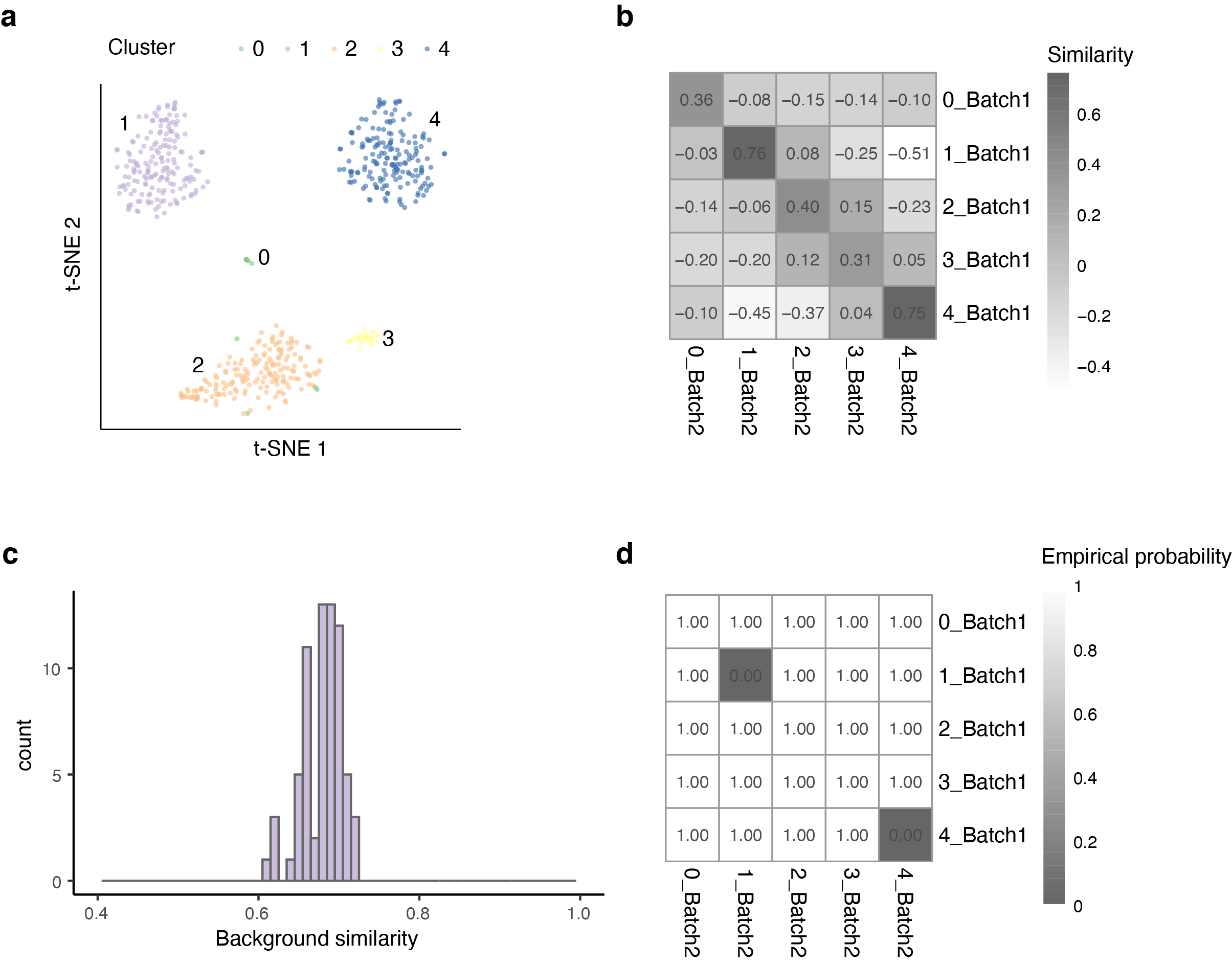


**Fig S7. CIDER evaluates integration results by identifying misaligned populations. (a)** *t*-SNE plot shows DBSCAN results of CCA-corrected dendritic data. **(b)** The IDER-based similarity between initial clusters from two batches. **(c)** The background distribution, i.e., the distribution of IDER-based similarity within the positive control groups that have the highest similarity. Here 1_Batch1 and 1_Batch2 are used. **(d)** The empirical probability of rejection.

**Table S1: Summarized information of datasets.**

| ID | Data type | Cell numbers | Purpose | Features |
| --- | --- | --- | --- | --- |
| Dataset 1 | Cell line | 9530 | Proof-of-concept experiment | Non-overlap |
| Dataset 2 | Simulation | 6000 | Benchmark clustering performance | Non-overlap |
| Dataset 3 | PBMC | 14876 | Benchmark clustering performance | Different platforms |
| Dataset 4 | Pancreas | 10127 | Benchmark clustering performance | Cross-species |
| Dataset 5 | PBMC from COVID-19, severe influenza and control conditions | 59572 | Benchmark clustering performance | Diseases |
| Dataset 6 | Breast cancer | 170350 | Benchmark clustering performance | Large dataset, inconsistent heterogeneity |
| Dataset 7 | Dendritic | 564 | Evaluation | Non-overlap |
| Dataset 8 | Mouse hematopoietic progenitors | 1442 | Evaluation | Trajectory |
