# Supplementary material for: CIDER: an interpretable meta-clustering framework for single-cell RNA-seq data integration and evaluation
Source: Genome Biol. 2021 Dec 13;22:337. doi: 10.1186/s13059-021-02561-2 (PMC8667531; doi:10.1186/s13059-021-02561-2)
Supplement: Supplementary file 2 — Additional file 2. Review history. [file 13059_2021_2561_MOESM2_ESM.docx]

Review History

**First round of review**

**Reviewer 1**

**Were you able to assess all statistics in the manuscript, including the appropriateness of statistical tests used?**

Yes: No additional statistical review is required.

**Were you able to directly test the methods?**

No.

**Comments to author:**

This paper presents a meta-clustering algorithm, CIDER, that perform clustering and integration of scRNA-seq across multiple batches. The main claim is that CIDER outperforms other scRNA-seq clustering methods and integration approaches on simulated and real data. The core idea behind this method was based on a previous paper by this group (ref [10]). In this paper, they compared two variants of their methods (asCIDER and dcCIDER) against seven analysis pipelines (CCA, MNN, Scanorama, Seurat, SC3, and RaceID) using 6 real public data sets and 1 simulated data set (by Splatter).

The idea is intuitive and simple, but the evaluation data seem deficient to support their rather strong claim that 'CIDER outperforms other scRNA-seq clustering methods and integration approaches'. Here are my main concerns:

1. Nowadays there are many excellent clustering and batch effect removal algorithms, so I am surprised that a relatively small number of algorithms were tested and compared. The simulation data (Supp Fig 3) look quite simplistic as they consist of mostly simple 'ball-shaped' clusters.

2. Many data sets seem relatively small (See Supp Table 1), with only 1 data set (Dataset 5) having more than 20,000 cells. In fact, given the motivation of tackling the problem of integrating single cell data from different patients (Abstract), I am surprised that, beside blood cells in which the cell types are often more well defined, there are basically no clinical samples from solid tissues from patients, such as tumours from cancer patients.

3. Looking at Dataset 5 (the largest data set with ~60K cells), the runtime of CIDER is in the order of 2000-4000 seconds, whereas the commonly used methods such as Seurat and Harmony finished within 100 seconds. In many clinical sample integration projects, we are often faced with integration of 10-20 samples, each having 10,000 cells, so a useful pipeline should be able to handle large data sets well. In this case, it is unclear if CIDER can indeed be scalable to larger data sets. In the main text, the authors use the term 'comparable' without qualifying whether the runtime was faster or slower than the comparator algorithms (line 6 of page 9 of manuscript). This may misguide the readers.

4. Several different evaluation criteria, such as ARI_population, ARI_batch, LISI, etc, were shown in different places in the manuscript and the supplementary materials. The authors should ideally apply all the evaluation metric on all the data sets across all them methods, and display the results in a consistent manner. At the moment, it is not easy to fully understand the evaluation results.

5. I find the term 'interpretable' in the title highly questionable. This claim originates from the idea that CIDER make use of differentially expressed genes to help align the clusters across batches. I cannot see why this necessarily make the framework or the results more 'interpretable'?

6. The R scripts are available via github, but it would be much better if the code can be turned into an R package that can be easily installed from github or bioconductor directly. The current README file is not informative.

Minor concerns:

1. The reference 'Efficient integration of heterogeneous single-cell transcriptomes using Scanorama' has appeared many times in the reference list (6, 15, 16, etc).

2. The reference 'Massively parallel digital transcriptional profiling of single cells' appeared several times

3. Some references have no author names.

**Reviewer 2**

**Were you able to assess all statistics in the manuscript, including the appropriateness of statistical tests used?**

No.

**Were you able to directly test the methods?**

No.

**Comments to author:**

The authors proposed a meta-clustering framework CIDER via a new metric to measure the similarity between cell groups. Those cell groups need to be independently identified from each scRNA-seq dataset. The goal of the method is to link the cell groups among multiple scRNA-seq datasets and predict the shared and dataset-specific cell groups. However, the proposed method is only able to measure the similarity between cell groups, and it lacks the capability of joint analysis in an integrated space. This makes the method much less useful because it is often critically important to integrate multiple datasets for a joint analysis of cell states, pseudotemporal trajectory, and other downstream tasks. Overall, it's unclear what significant advances the proposed method provides compared to many existing methods. Below are specific comments.

Comments:

1. CIDER cannot provide batch corrected data or compute a joint low-dimensional space for downstream analyses such as state identification and pseudotemporal analysis.

2. As initial clustering is needed for dnCIDER. What is the difference between the initial clustering and final clustering result? A comparison using both visualization and metrics is important.

3. The authors provided a strategy to evaluate the similarity between clusters from different conditions using differentially expressed gene signatures. How to determine whether the clusters represent the same cell type? How to select the threshold value of the similarity score? And how about the robustness of the threshold value?

4. The manuscript didn't provide much downstream analyses, such as joint visualization and pseudotime inference, making it difficult to evaluate the quality of the method in comparing with other methods.

**Reviewer 3**

**Were you able to assess all statistics in the manuscript, including the appropriateness of statistical tests used?**

Yes.

**Were you able to directly test the methods?**

Yes.

**Comments to author:**

The authors present an exciting new approach to (meta-)clustering of single-cell RNA-seq data that tackles the clustering challenge in a new way, based on a novel similarity metric defined from differential expression signatures of clusters across batches. CIDER is impressive in its ability to: 1) cluster cells; 2) integrate data across batches/individuals/etc; and 3) evaluate the quality of data integration without the need for ground-truth labels. I find the results shown support the claimed results well and overall the paper is well written and clear. With an efficient and user-friendly implementation (which from my reading could be missing), CIDER promises to be a very useful addition to the data analysis toolkit for scRNA-seq data.

Major:

- Credit to the authors for making their analysis scripts available on GitHub ([https://github.com/zhiyhu/CIDER-paper](https://urldefense.proofpoint.com/v2/url?u=https-3A__github.com_zhiyhu_CIDER-2Dpaper&d=DwQF-g&c=vh6FgFnduejNhPPD0fl_yRaSfZy8CWbWnIf4XJhSqx8&r=Y4r1_XDXu4yYdegfBIAiGEOf3a2Qz0JY3Z5HybxTDto&m=U58k5eHZBwLceGzH723vz_9q_4CbMASeeN2mUQHW9xc&s=AKxSKHLzdf9ikxuzgsylt82ny2Pl-kWKeU3pz3ddUQo&e=)). However, I can't see any reference to the availability of a software package implementing the CIDER methods so that others can use them. CIDER should be made available as a Bioconductor package, making use of the Bioconductor data structures and conventions to integrate it fully with the Bioconductor ecosystem for single-cell RNA-seq data analysis (along with the high code testing and documentation standards expected of Bioc packages).

- The paper would benefit from an explicit discussion of the scalability of the CIDER method(s) - results in Supp Fig 7 show that it may become computationally infeasible to run on very large datasets (for a given value of very large). It would be helpful as a reader to have guidance on when CIDER is expected to perform well in a "reasonable" amount of time (subjective, of course) and when other methods or strategies for using CIDER might be necessary.

Qs (partly for my interest, but mainly the paper would be improved if the following could be answered):

- If multiple patients/individuals/"experimental/observational units" are present within a dataset/batch, should clustering be done within batch AND patient? i.e. what is the correct level at which to do the "within dataset" clustering? (Relevant to Methods on p15, which could be expanded to answer these questions explicitly for the reader)

- Why is there such a difference in runtime for the CIDER methods between Dataset 3 and Dataset 4 (from near-fastest to middle-of-the-pack)? What characteristics of a dataset determine the runtime of the CIDER methods?

Minor:

- I love the network/similarity plots between clusters/cell types across batch/dataset. Very insightful. (Adding a legend defining the strength of similarity indicated by edge width would improve them even further.)

- Labels in Fig 2g are too small to read easily (even on a large screen trying to zoom in); some issues with numbers in Fig 2f too.

- Fig 2h - mention in the caption what Dataset 5 is (briefly, to save scrolling to Methods).

- p8 lines 20-21: reference to Fig 5f, but that doesn't exist - must refer to Fig 2f, I think?

- Would help with interpretation of "high-agreement genes" to point out explicitly that high-agreement genes should have small residuals (unless they have large residuals, in which case you should *definitely* state it explicitly as I have misunderstood); without further discussion I think it's hard for the reader to interpret Fig 2g as showing a lack of high-agreement genes.

- The IDER Methods paragraph on p14 lines 18-21 makes sense to an expert reader, but I think it would be worth fleshing out the explanation further by giving a concrete example or two of how the IDER calculation can be done with additional confounding factors/covariates. That would help all readers understand how broadly IDER can be applied.

- The explanation of the workflow for evaluating integration is a little hard to follow - an explanatory figure would surely make the approach much clearer to the reader.

- A more detailed description of LISI would be very helpful in the Methods on p19.

- An (incomplete?) reference for Scanorama appears at least seven times in the reference list???

- There style/formatting inconsistencies between entries in the reference list.

Typos:

- "IDEr" in Fig 1 caption.

- p6 line 16: Should refer to Supp Fig S3?

- p6 line 17: should be "where three batches were composed..."?

- p8 line 4: need an "and" after "(d)"?

- p6 line 21: should refer to Supp Fig 3c, e?

- p6 line 25: should refer to Fig 2c? [I might need to stop pointing these out...please just closely check all Fig refs].

- There are a bunch of typos in the captions of the Supp Figs (e.g. "abstract of residuals" should be "absolute value of residuals") - please just go through very carefully to correct throughout.

- p9 line 17: "has" -> "have".

- p11 line 20: "ancestry" -> "ancestor".

- Final sentence on p12 reads a little weirdly; I'd reword for clarity and elegance.

- p13 line 10: "genes level" -> "gene level".

- p13 line 18: "using negative binomial" -> "using a negative binomial".

- p13 line 21: "report" -> "results".

- p14 line 4: "compare" -> "compares".

- p17 line 6: "Scanorama-correct" -> "Scanorama-corrected".

- p17 line 19: "Scater object" -> more correct would be "SingleCellExperiment object".

**Authors Response**

**Point-by-point responses to the reviewers’ comments:**

Reviewer #1:

This paper presents a meta-clustering algorithm, CIDER, that perform clustering and integration of scRNA-seq across multiple batches. The main claim is that CIDER outperforms other scRNA-seq clustering methods and integration approaches on simulated and real data. The core idea behind this method was based on a previous paper by this group (ref [10]). In this paper, they compared two variants of their methods (asCIDER and dcCIDER) against seven analysis pipelines (CCA, MNN, Scanorama, Seurat, SC3, and RaceID) using 6 real public data sets and 1 simulated data set (by Splatter).

The idea is intuitive and simple, but the evaluation data seem deficient to support their rather strong claim that 'CIDER outperforms other scRNA-seq clustering methods and integration approaches'. Here are my main concerns:

1. Nowadays there are many excellent clustering and batch effect removal algorithms, so I am surprised that a relatively small number of algorithms were tested and compared. The simulation data (Supp Fig 3) look quite simplistic as they consist of mostly simple 'ball-shaped' clusters.

*Reply: We agree with the referee that there are many emergent well-working algorithms in the related topics. We have added 5 more integration methods (14 methods in total) into the benchmarking and showed that CIDER is still in the leading position considering the correctness of cell assignment and the scalability (new Figures 2-6).*

*We agree that though the simulation data seems artificial they still act as effective example tests for overcorrection illustrating the concept – in this case the potential clustering biases caused by confounders. Additionally, we have shown the performance of CIDER with four real datasets (Figures 3-6). We believe the comparison results to be sufficiently comprehensive to demonstrate the advantages of our methods and all our analysis code is available for reproduction of our results.*

2. Many data sets seem relatively small (See Supp Table 1), with only 1 data set (Dataset 5) having more than 20,000 cells. In fact, given the motivation of tackling the problem of integrating single cell data from different patients (Abstract), I am surprised that, beside blood cells in which the cell types are often more well defined, there are basically no clinical samples from solid tissues from patients, such as tumours from cancer patients.

*Reply: We thank the reviewer for the suggestion. We have now benchmarked CIDER on a breast cancer dataset (Dataset 6) containing 170,350 cells from 31 patients with the estrogen receptor-positive (ER+) subtype, the human epidermal growth factor receptor 2-negative (HER2-) subtype and the triple-negative breast cancer (TNBC) and demonstrated that CIDER methods not only had higher accuracy in identifying across-donor populations (Fig. 6a) but also consumed less time than other integration methods applied to the full dataset (Fig. 6c).*

3. Looking at Dataset 5 (the largest data set with ~60K cells), the runtime of CIDER is in the order of 2000-4000 seconds, whereas the commonly used methods such as Seurat and Harmony finished within 100 seconds. In many clinical sample integration projects, we are often faced with integration of 10-20 samples, each having 10,000 cells, so a useful pipeline should be able to handle large data sets well. In this case, it is unclear if CIDER can indeed be scalable to larger data sets. In the main text, the authors use the term 'comparable' without qualifying whether the runtime was faster or slower than the comparator algorithms (line 6 of page 9 of manuscript). This may misguide the readers .

*Reply: We have significantly improved the scalability of CIDER by optimizing the code base and adapting the multi-threading computing. Additionally, as described in the answer to Comment #2, we benchmarked CIDER on a large breast cancer dataset with 170,350 cells from 31 patients. The runtime of CIDER was shorter than many other integration methods that could cope with full-size data and - in absolute terms – never impractically slower than the fastest methods. We have also provided the exact values of runtime and other metrics in the figures to avoid misleading the readers. We thank the reviewer for encouraging us to improve the efficiency and scalability of the method.*

4. Several different evaluation criteria, such as ARI_population, ARI_batch, LISI, etc, were shown in different places in the manuscript and the supplementary materials. The authors should ideally apply all the evaluation metric on all the data sets across all them methods, and display the results in a consistent manner. At the moment, it is not easy to fully understand the evaluation results.

*Reply: We have reorganized Figures 2-6 for consistent visualization of comparison results.*

5. I find the term 'interpretable' in the title highly questionable. This claim originates from the idea that CIDER make use of differentially expressed genes to help align the clusters across batches. I cannot see why this necessarily make the framework or the results more 'interpretable'?

*Reply: We have elucidated the concept of interpretability in the Background section:*

*“Further, it is often difficult to determine why existing methods drive cells from different batches into the same cluster. This lack of explainability or interpretability can make it difficult to ascertain if integration has been successful.”*

*We have also expounded the interpretability of CIDER with the example in the pancreas dataset as follows:*

*“Unlike methods based on low-dimensional space, the gene-level analysis of CIDER empowered its explainability by delineating how various genes contributed to inter-group similarity. The influence of individual genes was derived by the Fisher z-transformation. Positive values of influence indicated the affirmative contribution to similarity, while negative values denoted the contribution to dissimilarity. For example, the inter-species similarity (0.40) of the ductal cell population was suppressed by the existence of negative-influence genes, e.g., CLU, TMSB4X and B2M (Fig. 4e). Yet the top positive-influence genes, e.g., KRT8 and KRT18, were the main drivers of aligning human and mouse ductal groups. On the other hand, the alpha cell population had a high value of inter-species similarity (0.62) owing to top positive-influence genes, e.g., GCG and TTR (Fig. 4f).”*

6. The R scripts are available via github, but it would be much better if the code can be turned into an R package that can be easily installed from github or bioconductor directly. The current README file is not informative.

*Reply: We thank the reviewer for this suggestion and encouragement. We have developed the R package and deployed it on GitHub. This package can be downloaded from GitHub (https://github.com/zhiyhu/CIDER/). We have also added documents (a Readme file and four vignettes), which can be viewed on the home page of this package: https://zhiyhu.github.io/CIDER/. Should this work be acceptable for publication and no further changes are required, we will then submit to Bioconductor.*

Minor concerns:

1. The reference 'Efficient integration of heterogeneous single-cell transcriptomes using Scanorama' has appeared many times in the reference list (6, 15, 16, etc).

*Reply: We thank the reviewer for the suggestion on the reference list. This bibliographical error has been corrected.*

2. The reference 'Massively parallel digital transcriptional profiling of single cells' appeared several times

*Reply: This bibliographical error has been corrected.*

3. Some references have no author names

*Reply: This bibliographical error has been corrected.*

Reviewer #2:

The authors proposed a meta-clustering framework CIDER via a new metric to measure the similarity between cell groups. Those cell groups need to be independently identified from each scRNA-seq dataset. The goal of the method is to link the cell groups among multiple scRNA-seq datasets and predict the shared and dataset-specific cell groups. However, the proposed method is only able to measure the similarity between cell groups, and it lacks the capability of joint analysis in an integrated space. This makes the method much less useful because it is often critically important to integrate multiple datasets for a joint analysis of cell states, pseudotemporal trajectory, and other downstream tasks. Overall, it's unclear what significant advances the proposed method provides compared to many existing methods. Below are specific comments.

Comments:

1. CIDER cannot provide batch corrected data or compute a joint low-dimensional space for downstream analyses such as state identification and pseudotemporal analysis.

*Reply: We present CIDER as a framework purposed for joint clustering without relying on a “corrected” low-dimensional space. It is an advantage of CIDER given the growing homogeneity of tools in this field.*

*The output of CIDER is the cell assignment, i.e., identification of cell types, subtypes and states. As it has incorporated the clustering function, it does not need an additional step of downstream analysis for state identification. To clarify, we added the visualization of CIDER clustering results in Fig. 3d, e.*

*We exemplify the downstream analysis for marker gene analysis, and the results were presented in Fig. 3f, g.*

*Like other clustering methods, we recommend that CIDER is used with data where there is a latent discrete population structure. It is not recommended to use the clustering function of CIDER for pseudotemporal analysis on data with a continuous manifold. However, we did test the function of CIDER in this scenario, and it can be used for continuous trajectories as demonstrated in mouse hematopoietic progenitor data (Dataset 8; Fig. 9). However, we also state in the Discussion that further specific methodological development maybe required to optimize this application.*

2. As initial clustering is needed for dnCIDER. What is the difference between the initial clustering and final clustering result? A comparison using both visualization and metrics is important.

*Reply: The meta-clustering concept used by CIDER can be interpreted as two-tier clustering. As described in the Methods section, “the initial clusters were merged by the hierarchical agglomerative clustering with complete linkage”, which generated the clustering results, i.e., final clusters.*

*In the revised manuscript, we presented the initial clustering results, final clustering results and their relationships in Fig. S4c, Fig. 3d&e and Fig. S4d, respectively.*

*Last but not the least, as we have incorporated CIDER into an R package, the initial clusters and final clusters are both kept in the metadata of the Seurat object, which can be visualized or compared depending on the users’ demand.*

3. The authors provided a strategy to evaluate the similarity between clusters from different conditions using differentially expressed gene signatures. How to determine whether the clusters represent the same cell type? How to select the threshold value of the similarity score? And how about the robustness of the threshold value?

*Reply: The similarity measurement of CIDER quantifies the inter-group distances using a continuous spectrum, which mimics real-world scenarios. We agree with the reviewer that this continuous measure needs to be dichotomized for human interpretation.*

*To help decide the threshold of similarity, we introduced the empirical probability of rejection as follows:*

*“Empirical probability of rejecting the alignment is next computed by comparing the distributions of similarity between the targeted cluster and the background. Low similarity or high empirical probability putatively indicate the falsely aligned cluster, i.e., rejection of the fact that cells from a cross-batch cluster belong to a homogeneous population.”*

*As demonstrated in the example of the dendritic dataset (Fig. 8), the test metrics computed by CIDER could accurately identify the correctly aligned clusters (empirical probability of rejection = 0) and the falsely aligned clusters (empirical probability of rejection = 1).*

4. The manuscript didn't provide much downstream analyses, such as joint visualization and pseudotime inference, making it difficult to evaluate the quality of the method in comparing with other methods.

*Reply: Our method, a meta-clustering framework, is aimed to address the classification task of identifying cell populations for confounded data.*

*We have compared CIDER with other methods using metrics of ARIpopulation (accuracy of cell assignment), 1-ARIbatch (efficiency of removing unwanted variables) and runtime (scalability) and demonstrated the superiority of our methods.*

*We have added examples of more downstream analysis as elucidated in the answer to Comment #1.*

Reviewer #3:

The authors present an exciting new approach to (meta-)clustering of single-cell RNA-seq data that tackles the clustering challenge in a new way, based on a novel similarity metric defined from differential expression signatures of clusters across batches. CIDER is impressive in its ability to: 1) cluster cells; 2) integrate data across batches/individuals/etc; and 3) evaluate the quality of data integration without the need for ground-truth labels. I find the results shown support the claimed results well and overall the paper is well written and clear. With an efficient and user-friendly implementation (which from my reading could be missing), CIDER promises to be a very useful addition to the data analysis toolkit for scRNA-seq data.

Major:

- Credit to the authors for making their analysis scripts available on GitHub (https://github.com/zhiyhu/CIDER-paper). However, I can't see any reference to the availability of a software package implementing the CIDER methods so that others can use them. CIDER should be made available as a Bioconductor package, making use of the Bioconductor data structures and conventions to integrate it fully with the Bioconductor ecosystem for single-cell RNA-seq data analysis (along with the high code testing and documentation standards expected of Bioc packages).

*Reply: We thank the reviewer for this suggestion. We have developed the R package and deployed it on GitHub. This package can be downloaded from GitHub (https://github.com/zhiyhu/CIDER/). We have also added detailed documents and tutorials (a Readme file and four Rmarkdown vignettes) which can be viewed on the home page of this package: https://zhiyhu.github.io/CIDER/. We are ready to submit to Bioconductor but would like to see the manuscript be accepted for publication and that no more substantive changes to the software are required before doing so.*

- The paper would benefit from an explicit discussion of the scalability of the CIDER method(s) - results in Supp Fig 7 show that it may become computationally infeasible to run on very large datasets (for a given value of very large). It would be helpful as a reader to have guidance on when CIDER is expected to perform well in a "reasonable" amount of time (subjective, of course) and when other methods or strategies for using CIDER might be necessary.

*Reply: We have significantly improved the scalability of CIDER by optimizing the code base and adapting the multi-threading computing. Additionally, we benchmarked CIDER on a large dataset with 170,350 cells from 31 patients, in which the runtime of CIDER was shorter than many other integration methods that could cope with full-size data and in absolute terms not impractically slower than the fastest methods.*

Qs (partly for my interest, but mainly the paper would be improved if the following could be answered):

- If multiple patients/individuals/"experimental/observational units" are present within a dataset/batch, should clustering be done within batch AND patient? i.e. what is the correct level at which to do the "within dataset" clustering? (Relevant to Methods on p15, which could be expanded to answer these questions explicitly for the reader)

*Reply: We thank the reviewer for this suggestion. The answer to the questions has been added to the Discussion as follows:*

*“A common question of integration is which effects should be considered. Two criteria, the magnitude of the bias and their relevance to the purpose of the study, can be used to choose covariates for correction. In the first scenario, such as Dataset 2 (simulated data) and Datasets 3 (PBMC), the simulated batch effect and the technical effect introduced bias into the clustering if not corrected (Fig. 2c, 3a), indicating the covariates for regression. On the other hand, advances in the droplet-based scRNA-Seq platform and the cryopreservation technique have enabled the minimization of technically introduced batch effects. Thus, in the experiments that follow one consistent experimental protocol and include multiple donors, the inter-sample variability can be largely attributed to the “biological” variability, such as donors’ condition and genetic diversity [27,28]. In this scenario, the selection of covariates for regression can be based on the relevance to the research goal. For example, the health condition in Dataset 5 (COVID-19 versus severe flu) and the donor, as well as the treatment, in Dataset 6 (breast cancer) was corrected to identify cross-condition and cross-donor populations.”*

- Why is there such a difference in runtime for the CIDER methods between Dataset 3 and Dataset 4 (from near-fastest to middle-of-the-pack)? What characteristics of a dataset determine the runtime of the CIDER methods?

*Reply: We have added description in the Results section:*

*“Dataset 4 (n = 10,127) has fewer cells than Dataset 3 (n = 14,876). CIDER took longer time to process Dataset 4 than Dataset 3, because the running time of CIDER methods was approximately associated with the numbers of batch-specific clusters.”*

*We also added the explanation in the Methods section:*

*“Given that CIDER needed to compute pairwise similarity, the runtime of CIDER was approximately O(n^2), where n denotes the number of batch-specific initial clusters. It was also positively associated with the covariates and the number of genes included in the regression.”*

Minor:

- I love the network/similarity plots between clusters/cell types across batch/dataset. Very insightful. (Adding a legend defining the strength of similarity indicated by edge width would improve them even further.)

*Reply: We appreciate the reviewer’s recognition of our visualization methods. The legend has been added to the network graph (Fig. 2b, S4d).*

*- Labels in Fig 2g are too small to read easily (even on a large screen trying to zoom in); some issues with numbers in Fig 2f too.*

*Reply: Old Fig. 2f, g has been moved to Fig. 4d, e and the font has been increased for clarity.*

- Fig 2h - mention in the caption what Dataset 5 is (briefly, to save scrolling to Methods)

*Reply: The description has been added to the caption of new Figure 5: “Figure 5. CIDER identified cross-condition cell classes in the PBMC data from healthy donors and patients with COVID-19 or influenza (Dataset 5).”*

- p8 lines 20-21: reference to Fig 5f, but that doesn't exist - must refer to Fig 2f, I think?

*Reply: We have corrected it in the revised manuscript.*

- Would help with interpretation of "high-agreement genes" to point out explicitly that high-agreement genes should have small residuals (unless they have large residuals, in which case you should *definitely* state it explicitly as I have misunderstood); without further discussion I think it's hard for the reader to interpret Fig 2g as showing a lack of high-agreement genes.

*Reply: We appreciate the reviewer’s suggestion. We have improved the approach to quantifying the influence of individual genes on inter-batch similarity by using the Fisher z-transformation.*

*We have updated the figure (new Fig. 4e-f) and the Results section to further elucidate this point:*

*“Unlike methods based on low-dimensional space, the gene-level analysis of CIDER empowered its explainability by delineating how various genes contributed to inter-group similarity. The influence of individual genes was derived by the Fisher z-Transformation. Positive values of influence indicated the affirmative contribution to similarity, while negative values denoted the contribution to dissimilarity. For example, the inter-species similarity (0.40) of the ductal cell population was suppressed by the existence of negative-influence genes, e.g., CLU, TMSB4X and B2M (Fig. 4e). Yet the top positive-influence genes, e.g., KRT8 and KRT18, were the main drivers of aligning human and mouse ductal groups. On the other hand, the alpha cell population had a high value of inter-species similarity (0.62) owing to top positive-influence genes, e.g., GCG and TTR (Fig. 4f).”*

- The IDER Methods paragraph on p14 lines 18-21 makes sense to an expert reader, but I think it would be worth fleshing out the explanation further by giving a concrete example or two of how the IDER calculation can be done with additional confounding factors/covariates. That would help all readers understand how broadly IDER can be applied.

*Reply: We thank the reviewer for the suggestion. We have included a new dataset (Dataset 6, Fig. 6) to explicate the scenario of multiple covariates as follows.*

*“To demonstrate the scalability of CIDER, we benchmarked CIDER and other methods on a breast cancer dataset (Dataset 6) containing 170,350 cells from 31 patients with the estrogen receptor-positive (ER+) subtype, the human epidermal growth factor receptor 2-negative (HER2-) subtype and the triple-negative breast cancer (TNBC) [23]. For each patient, two samples were collected, one before the treatment and one during the subsequent surgery. Thus, three potential covariates existed, namely the donor effect, the treatment effect and the disease effect, and donor was the nested variable to disease. To identify the cross-patient populations, we generated patient-specific initial clusters and then used donor and treatment (pre-treatment or on-treatment) as covariates to calculate the IDER-based similarity matrix, which enabled regressing donor and treatment effects.”*

- The explanation of the workflow for evaluating integration is a little hard to follow - an explanatory figure would surely make the approach much clearer to the reader

*Reply: A diagram elucidating the workflow for evaluating integration has been added to the new Fig. 7b.*

- A more detailed description of LISI would be very helpful in the Methods on p19.

*Reply: The description has been added to the Methods section as follows:*

*“The cLISI metric, computed by R package lisi [11], was used to validate the similarity and the empirical probability calculated by CIDER. LISI measures the population diversity within the neighbors of a given cell, and the neighborhoods are defined by Gaussian kernel-based distributions. Here cLISI was calculated as the LISI between the ground-truth annotations of cell populations in the batch-corrected t-SNE space.”*

- An (incomplete?) reference for Scanorama appears at least seven times in the reference list???

*Reply: The reference for the Scanorama has been fixed.*

- There style/formatting inconsistencies between entries in the reference list.

*Reply: This bibliographical error has been corrected.*

Typos:

- "IDEr" in Fig 1 caption.

*Reply: Corrected.*

- p6 line 16: Should refer to Supp Fig S3?

*Reply: Corrected.*

- p6 line 17: should be "where three batches were composed..."?

*Reply: Corrected.*

- p8 line 4: need an "and" after "(d)"?

*Reply: No longer need to correct.*

- p6 line 21: should refer to Supp Fig 3c, e?

*Reply: Corrected.*

- p6 line 25: should refer to Fig 2c? [I might need to stop pointing these out...please just closely check all Fig refs].

*Reply: Corrected.*

- There are a bunch of typos in the captions of the Supp Figs (e.g. "abstract of residuals" should be "absolute value of residuals") - please just go through very carefully to correct throughout.

*Reply: Corrected.*

- p9 line 17: "has" -> "have".

*Reply: Corrected.*

- p11 line 20: "ancestry" -> "ancestor".

*Reply: Corrected.*

- Final sentence on p12 reads a little weirdly; I'd reword for clarity and elegance*.*

*Reply: Modified.*

- p13 line 10: "genes level" -> "gene level".

*Reply: Corrected.*

- p13 line 18: "using negative binomial" -> "using a negative binomial".

*Reply: Corrected.*

- p13 line 21: "report" -> "results".

*Reply: Corrected.*

- p14 line 4: "compare" -> "compares".

*Reply: Corrected.*

- p17 line 6: "Scanorama-correct" -> "Scanorama-corrected".

*Reply: Corrected.*

- p17 line 19: "Scater object" -> more correct would be "SingleCellExperiment object".

*Reply: Corrected.*

**Second round of review**

**Reviewer 1**

The manuscript and the software package have been significantly revised. Most of my concerns regarding validity and comprehensiveness of the evaluation have been addressed. The github code works. Multi-threading improves the runtime performance of their code substantially. To make the tool useful to a wide userbase, I hope the authors will really submit their package to bioconductor. I think the primary value of this paper is its tool.

**Reviewer 2**

I am fully satisfied with the revisions the authors have made in the revised manuscript. Congratulations on thorough revisions and an excellent paper.

**Authors Response**

**Point-by-point responses to the reviewers’ comments:**

The manuscript and the software package have been significantly revised. Most of my concerns regarding validity and comprehensiveness of the evaluation have been addressed. The github code works. Multi-threading improves the runtime performance of their code substantially. To make the tool useful to a wide userbase, I hope the authors will really submit their package to bioconductor. I think the primary value of this paper is its tool.

*Reply: Thank you for the comments and support for publication. We have now:*

*1) Produced a CRAN package.*

*2) Provided a Zenodo reference for our Github repository.*

*3) Provided information on all datasets used as reqested.*

*4) Modified the article title as suggested.*

*5) Added Funding, and Ethical Approval sections.*

*6) Removed cover letter and responses document.*

*7) Removed the line numbers.*

*8) Uploaded all main figure files.*

*9) Made appropriate changes to Additional File.*
